# Recent Advances in Single-Atom Electrocatalysts for Oxygen Reduction Reaction

**DOI:** 10.34133/2020/9512763

**Published:** 2020-08-14

**Authors:** Junxing Han, Juanjuan Bian, Chunwen Sun

**Affiliations:** ^1^CAS Center for Excellence in Nanoscience, Beijing Institute of Nanoenergy and Nanosystems, Chinese Academy of Sciences, Beijing 100083, China; ^2^School of Nanoscience and Technology, University of Chinese Academy of Sciences, Beijing 100049, China

## Abstract

Oxygen reduction reaction (ORR) plays significant roles in electrochemical energy storage and conversion systems as well as clean synthesis of fine chemicals. However, the ORR process shows sluggish kinetics and requires platinum-group noble metal catalysts to accelerate the reaction. The high cost, rare reservation, and unsatisfied durability significantly impede large-scale commercialization of platinum-based catalysts. Single-atom electrocatalysts (SAECs) featuring with well-defined structure, high intrinsic activity, and maximum atom efficiency have emerged as a novel field in electrocatalytic science since it is promising to substitute expensive platinum-group noble metal catalysts. However, finely fabricating SAECs with uniform and highly dense active sites, fully maximizing the utilization efficiency of active sites, and maintaining the atomically isolated sites as single-atom centers under harsh electrocatalytic conditions remain urgent challenges. In this review, we summarized recent advances of SAECs in synthesis, characterization, oxygen reduction reaction (ORR) performance, and applications in ORR-related H_2_O_2_ production, metal-air batteries, and low-temperature fuel cells. Relevant progress on tailoring the coordination structure of isolated metal centers by doping other metals or ligands, enriching the concentration of single-atom sites by increasing metal loadings, and engineering the porosity and electronic structure of the support by optimizing the mass and electron transport are also reviewed. Moreover, general strategies to synthesize SAECs with high metal loadings on practical scale are highlighted, the deep learning algorithm for rational design of SAECs is introduced, and theoretical understanding of active-site structures of SAECs is discussed as well. Perspectives on future directions and remaining challenges of SAECs are presented.

## 1. Introduction

**Figure 1:**
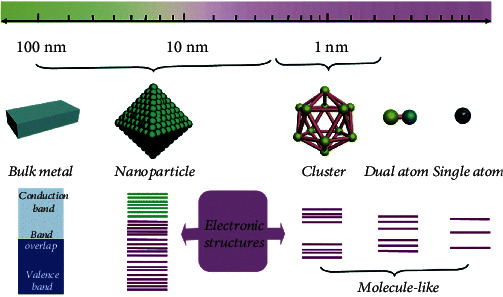
Electronic energy level changes in reducing the metal size. Reproduced from [4].

Energy plays crucial roles in the development of human civilization. Most energy-related process involves catalytic reactions by using catalysts to accelerate the reaction rate and elevate the product selectivity [1]. The phenomenon of catalysis has been recognized since ancient times, whereas the cognition of the essence merely began in the 18th century [2]. Catalysts are commonly divided into heterogeneous catalysts and homogeneous catalysts. Heterogeneous catalysts are composed of supported metal nanoparticles. Only a small part of metal atoms exposed on the surface participate in the reaction, whereas other metal atoms nonaccessible by the reactants are inert or even cause undesirable side reactions. Homogeneous catalysts possess tunable active-site structure, which enables them exhibiting ultrahigh activity and unique selectivity for specific reactions [3]. However, unsatisfactory stability and poor recyclability limit their applications. Coupling the merits of both homogeneous and heterogeneous catalysts is the ultimate goal in catalysis science. In this context, atomically isolated metal atoms embedded in suitable supports generate a new kind of catalysts, which patches up the gap between homogeneous and heterogeneous catalysis. The dispersion of metal on an atomic scale generates maximum utilization efficiency (nearly 100%), remarkable quantum size effect, unsaturated coordination environment, and strong metal-support interaction, as shown in Figure 1 [4], which enables metal atoms dramatically boosted activity, selectivity, and stability. Furthermore, recent progress in advanced characterization techniques makes it possible to confirm the exact geometric structure of the active centers due to their simplicity and homogeneity, which provides an ideal platform for establishing theoretical calculation models and deeply understanding catalytic mechanisms at the atomic-scale level to elucidate the fundamental synthesis-structure-performance correlations for rational design of catalysts for targeted reactions. This novel kind of catalysts was, for the first time, named as “single-atom catalysts (SACs)” by Qiao and coworkers in 2011 after precisely anchoring atomically isolated Pt atoms on FeO_*x*_ [5]. Since then, study on SACs experienced an explosive growth and SACs have been used in various reactions including propane dehydrogenation [6], *n*-hexane isomerization [7], C-H bond activation [8], Suzuki coupling [9], and CO oxidation [5]. Especially, the introduction of SACs in electrochemical reactions, including hydrogen evolution reaction (HER) [10], oxygen evolution reaction (OER) [11, 12], oxygen reduction reaction (ORR) [13], carbon dioxide reduction reaction (CO_2_RR) [14], and nitrogen reduction reaction (NRR) [15], boosts the great progress on synthesis, characterization, reaction mechanism, and structure-performance relationship of single-atom electrocatalysts (SAECs) due to the high detection sensitivity and low catalyst loadings for electrochemical reactions.

Among various electrochemical reactions catalyzed by SAECs, ORR plays significant roles in electrochemical energy storage and conversion technologies as well as clean synthesis of fine chemicals [16]. Hydrogen peroxide (H_2_O_2_) is regarded as one of the most widely used fine chemicals for water treatment and chemical synthesis [17]. The industrial demand for hydrogen peroxide has reached 4 million tons per year. The clean production of H_2_O_2_ through selectively electrochemical reduction of oxygen molecules involves the oxygen reduction reaction. The ideal catalysts for H_2_O_2_ synthesis should exhibit high ORR activity, high selectivity, and low reduction reaction activity toward H_2_O_2_. However, traditional bulk metal catalysts and metal nanoparticle catalysts show poor selectivity for H_2_O_2_ due to the cleavage of O-O bond by adjacent atom sites [18]. SAECs featuring with atomically isolated active sites promoted the adsorption of O_2_ adopting the end-on type other than peroxo configuration, lowering the probability of O-O bond cleavage. Therefore, SAECs would be an ideal catalyst for H_2_O_2_ production via ORR. Moreover, ORR is widely involved in the discharging process of various metal-air batteries, such as Li-O_2_ battery and zinc-air battery, as well as low-temperature fuel cells, like anion exchange membrane fuel cells (AEMFCs), proton exchange membrane fuel cells (PEMFCs), and microbial fuel cells (MFCs) [19]. The ORR rate significantly determines the output power density of these advanced electrochemical energy storage and conversion devices. Normally, the ORR process shows sluggish kinetics and requires platinum-group noble metal (PGNM) catalysts to accelerate the reaction. However, the scarce reservation, high cost, and poor durability significantly impede the large-scale commercialization of PGNM catalysts [20]. US Department of Energy (DOE) reported that PGNM electrocatalysts account for 55% cost of the fuel cell stack used for automobiles [21], barricading the commercialization of fuel cell technologies. To address these difficult problems, intensive efforts have been devoted to develop noble metal-free ORR catalysts with low cost and high activity in the past decades including metal-free carbon-based electrocatalysts [22], SAECs [23], metal nitride electrocatalysts [24], and metal alloy electrocatalysts [25]. Among these reported noble metal-free ORR catalysts, single-atom electrocatalysts (denoted as M-N_*x*_-C_*y*_, M=metal) show great potential to substitute PGNM catalysts because of their high intrinsic activity and tunable support structures [26].

Tremendous advantages and outstanding performance of SAECs in oxygen reduction reactions as well as ORR-related applications have intrigued substantial growth of research interest in the past several years [27]. In this review, recent advances of synthesis, characterization, ORR performance of SAECs, and their applications in ORR-related H_2_O_2_ production, metal-air batteries, and low-temperature fuel cells were systematically summarized. Relevant progresses on SAECs with binary metal centers, binary ligands, and unique architecture supports were also reviewed. For practical applications, general strategies to synthesize SAECs with high metal loadings on large scale are highlighted, the deep learning algorithm for highly efficient design of SAECs is introduced, and theoretical understanding of the active-site structure of SAECs and the structure-performance relationship is discussed. Finally, challenges and perspectives for SAECs are present to spotlight the future direction for further optimizing ORR performance.

## 2. Synthetic Methods for SAECs

SAECs are not directly available; in that, the individual metal atoms are prone to aggregate into nanoclusters or nanoparticles in the synthetic process to reduce surface energy. For the synthesis of SAECs, the following critical criteria should be considered: (i) uniform dispersion of active sites over the support, (ii) strong interaction between isolated metal atoms and adjacent coordination atoms, (iii) promotion of mass and electron transport as well as active-site accessibility, and (iv) high activity and durability. Various strategies for the synthesis of SAECs have been developed, including wet chemical methods, high-temperature pyrolysis methods, vapor deposition method, and atom trapping methods. For practical applications, some general methods for the synthesis of SAECs with high metal loadings on a large scale have also been developed.

### 2.1. Wet Chemical Methods

Wet chemical methods, including impregnation, modular coordination, colloidal strategy, electrochemical deposition, low-temperature reduction, and ion exchange, are of ease to operate and have been widely used for production on a large scale. The isolated metal atoms show strong interaction with the support, which ensures that the isolated metal atoms are dispersed on the surface of the support and prevents their aggregation. The key points to prepare high-performance SAECs include selecting suitable precursor materials and controlling the synthetic procedure.

#### 2.1.1. Impregnation

For impregnation method, the first and most important thing is to construct suitable anchoring sites to immobilize and disperse single-atom metal precursors. The commonly used anchoring sites include the defect sites on the metal oxides, the coordination atoms (such as B, N, P, O, S) doped in the carbon skeleton, the *π*-*π* interaction, and the covalent tethering.

By pyrolyzing a core shell-structured SiO_2_@melamine-resorcinol-formaldehyde polymer spheres, followed by HF etching, N-doped porous carbon spheres could be prepared. Then, a single-atom Co catalyst with CoN_5_ active sites was obtained by impregnating the N-doped carbon spheres into a cobalt phthalocyanine (CoPc) solution [33]. N atoms doped in the carbon matrix coordinated with the central Co atom of CoPc in the axial direction and firmly anchored the CoPc molecules on the carbon spheres. The same method could also be used to prepare Fe, Ni, and Cu single-atom catalysts by replacing CoPc with FePc, NiPc, and CuPc. Choi and coworkers prepared sulfur-doped zeolite-templated carbon (HSC) support with a high sulfur loading of 17 wt% [34]. By impregnating HSC into H_2_PtCl_6_ solution, the strong interaction between Pt and S could anchor isolated Pt atoms on the HSC support. After H_2_ reduction, a single-atom Pt/HSC catalyst with a high Pt loading of 5 wt% was successfully prepared. EXAFS and HAADF-STEM identified the generation of individual Pt^2+^ species coordinated with four S-moieties. By grinding sodium hypophosphite with the graphite carbon nitride (g-C_3_N_4_) nanosheets followed by thermal treatment at 300°C under inert atmosphere, Cao et al. prepared phosphor-decorated g-C_3_N_4_ nanosheets (PCN) [35]. By impregnating the obtained PCN in a RuCl_3_ solution at 70°C for 5 h, followed by thermal treatment at 300°C under argon atmosphere, a single-atom Ru catalyst (Ru-N-C) was prepared.

By mixing CoPc with CNTs in DMF associated with the assistance of magnetic stirring and sonication, CoPc molecules could be immobilized on the surface of CNTs to form the single-atom CoPc/CNT catalyst by the strong *π*-*π* interaction [36]. With the same method, amino functionalized CoPc (CoPc-NH_2_) could also be anchored onto the surface of CNTs to synthesize single-atom CoPc-NH_2_/CNT catalyst [37]. However, under harsh electrochemical reaction conditions, this kind of immobilization approach usually causes the exfoliation of the active CoPc molecules. Cao et al. reported a covalent tethered method to immobilized Fe phthalocyanine (FePc) onto single-walled carbon nanotubes (CNTs) to prepare single-atom Fe electrocatalyst (FePc-Py-CNTs) for ORR [38]. Firstly, purified CNTs were functionalized by reaction with 4-aminopyridine (4-AP) to prepare the Py-CNT support. Then, Py-CNTs were impregnated into a FePc solution and FePc molecules were tethered onto Py-CNTs by axial coordination. Compared with the *π*-*π* interaction, covalent tethering shows better stability.

In most cases, the synthesis of SAECs by impregnation is conducted in aqueous solutions, which limits the choice of precursors to water-soluble metal salts, preventing the use of organometallic metal salts containing organic ligands. Hutchings's group recently reported a facile synthesis of active carbon-supported precious metal-based (Pd, Pt, Ru, Au, etc.) single-atom catalysts in organic solvents with low-polarity and low-boiling-point solvent, such as ultradry acetone [39]. By simple impregnation, stabilized single cationic metal species were anchored on active carbon supports in the obtained single-atom catalysts. The substitution for water with organic solvents avoids the utilization of strongly oxidizing aqueous solutions and reduces the catalyst drying temperature, which prevent sintering of the highly dispersed metal species.

#### 2.1.2. Modular Coordination Method

**Figure 2:**
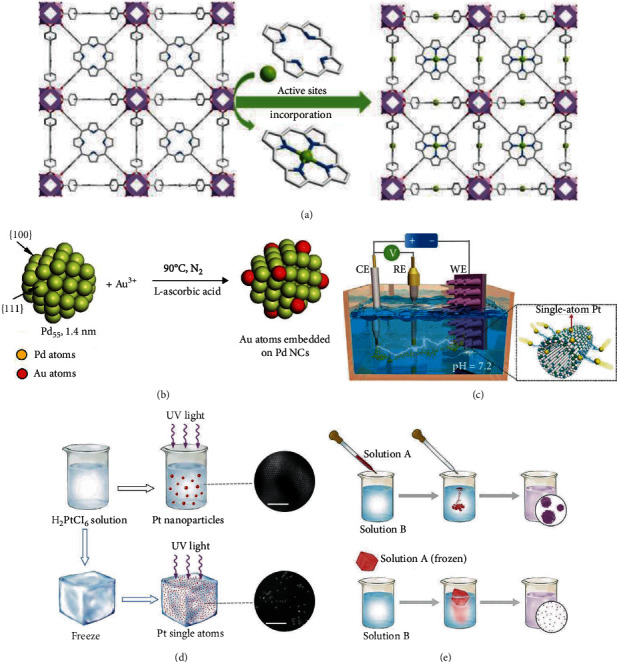
Wet chemical methods for the synthesis of SAECs. (a) Incorporation of active sites into a porous framework. Reproduced from [28]. (b) Schematic illustration of the deposition of single Au atoms on Pd mother clusters by the successive reduction method using L-ascorbic acid as the reducing reagent. Reproduced from [29]. (c) Schematic diagram of the electrochemical deposition of single-atom Pt onto CoP nanotubes. Reproduced from [30]. (d) Schematic illustration the iced-photochemical process for the preparation of atomically dispersed Pt catalysts. Reproduced from [31]. (e) Illustration of the syntheses of atomically dispersed metals. Reproduced from [32].

Metal organic frameworks (MOFs) consisting of organic linkers bonded with metal nodes show unique properties of tunable pore size, large specific surface area, and uniform structure. By incorporating porphyrin rings in the organic linkers, the obtained MOFs could be used as the host to coordinate with various metal cations due to the strong coordination properties of the porphyrin rings. The as-prepared materials show atomically dispersed single-atom metal sites with totally uniform structures and high loadings. The popularity of isolated metal atoms and the pore size of MOFs could be easily tuned by constructing different organic linkers. With zirconium chloride and tetrakis(4-carboxyphenyl) porphyrin as the precursor, a porphyrin-based MOF (MOF-525) was prepared [28]. Then, MOF-525 powders were immersed into a cobalt nitrate solution in DMF. The coordination of Co cations with the porphyrin rings in the MOF-525 matrix could anchor Co cations and form single-atom MOF-525-Co catalyst, as illustrated in Figure 2(a). In a similar way, a single-atom MOF-525-Zn catalyst was also prepared. For the first time, Sawano and coworkers synthesized a triaryl-phosphine-derived tricarboxylate linker, which was used to prepare a Zr-based MOF (P_1_-MOF) [40]. Postsynthetic metalation of P_1_-MOF with [Ir(OMe)(cod)]_2_ or [RhCl(nbd)]_2_ afforded single-atom P_1_-MOF-Rh or P_1_-MOF-Ir with controllable metal loadings. The strong coordination of isolated monophosphine sites in the P_1_-MOF matrix with metal precursors inhibits the mobilization of metal precursors and generates single-atom catalysts. With organic dicarboxylic acid and AlCl_3_ as the precursors, Zhou and coworkers synthesized a microporous MOF-253 [41]. By immobilizing a platinum complex (*cis*-Pt(DMSO)_2_Cl_2_) onto the 2,2′-bipyridine units, a single-atom MOF-253-Pt catalyst was prepared.

By replacing porphyrin with metalloporphyrin in the organic linkers, this kind of SAECs could be directly synthesized in one step. Zuo et al. reported a surfactant-stabilized coordination strategy towards two-dimensional single-atom Pt catalysts with a Pt loading as high as 12.0 wt% by embodying presynthesized Pt-porphyrin molecules into Cu-based MOF nanosheets [42]. This modular coordination is an ideal method to synthesize SAECs with uniform active-site structure and high metal loadings. However, the conductivity of the obtained SAECs is unsatisfactory, which inhibits the overall electrochemical catalytic efficiency.

#### 2.1.3. Colloidal Strategy

A series of colloidal single-atom catalysts were synthesized by embedding metal A atoms on another metal B nanoclusters with the successive reduction, simultaneous reduction, or galvanic replacement reactions. As illustrated in Figure 2(b), with the PVP-protected Pd nanoclusters as the mother support, the successive reduction of HAuCl_4_ produced a colloidal Au single-atom catalyst [29]. EELS measurements showed that Au clusters were located on several areas across the surface of the Pd_55_ nanoclusters. A golden single-atom Pt electrocatalyst (Pt_4_Au_96_) was prepared by reducing H_2_PtCl_6_ (4%) and HAuCl_4_ (96%) simultaneously in the mixture of oleylamine and ethylene glycol in one pot [43]. The individual Pt atoms were surrounded and stabilized by Au atoms of the bimetal nanocrystals. A PVP-protected colloidal isolated Au atom on Pd nanocluster catalyst was prepared by a galvanic replacement reaction method [44]. The Pd atoms at the top positions of Pd nanoclusters reacted with Au^3+^ ions and replaced by the formed Au atoms. This approach could also be used to synthesize trimetallic catalysts with isolated Au sites on IrPd bimetal nanoclusters [45].

#### 2.1.4. Electrochemical Deposition

MXenes, a new class of two-dimensional transition metal carbides/nitrides, feature exceptional properties: (i) excellent electronic conductivity with efficient charge transport, (ii) catalytically active basal planes with exposed metal sites, (iii) hydrophilic surface functionalities, and (iv) unique layered structure consisting of conductive transition metal carbides or nitrides. These attractive properties render MXenes as superior substrate for facilitating various electrocatalysts. For instance, electrochemically exfoliation Mxene nanosheets (Mo_2_TiC_2_T_*x*_) containing large quantities of exposed basal planes could generate abundant Mo vacancies, which could be used as the anchoring site to electrochemically deposit single-atom Pt [10].

As shown in Figure 2(c), Zhang et al. developed an electrochemical method to deposit single-atom Pt onto CoP nanotubes [30]. Firstly, they prepared a CoP nanotube arrays on Ni foams (NT-NF). Then, NT-NF, Pt foil, and saturated calomel electrode were assembled in a standard three-electrode filling with N_2_-saturated 1 M phosphate-buffered solution. After 5000 potential cycles, the single-atom PtSA-NT-NF electrocatalyst was obtained.

Very recently, Zhang et al. developed a general electrochemical deposition strategy, which could be applied in a wide range of metals and supports for the preparation of SAECs [46]. The deposition process was carried out on both anode and cathode. A glassy carbon electrode loaded with Co(OH)_2_ nanosheets was used as the working electrode and a diluted H_2_IrCl_6_ (100 *μ*M) solution was used as the metal precursor and the electrolyte. The depositing process started from 0.10 to -0.40 V in cathodic deposition and from 1.10 to 1.80 V in anodic deposition. The scanning cycle was repeated for three times to obtain A-Ir_1_/Co(OH)_2_ from the anode and ten times to obtain C-Ir_1_/Co(OH)_2_ from the cathode. More than 30 kinds of different SAECs (Fe, Ni, Co, Zn, Cu, Cr, V, Ag, Mn, Ru, Ir, Pd, Rh, Pt, Au, etc.) are prepared from anodic or cathodic deposition by changing supports and metal precursors.

#### 2.1.5. Low-Temperature Reduction Method

The ice lattice possesses natural confining effect. If an aqueous solution is frozen rapidly at ultralow temperature, an ice layer containing a homogeneous concentration of isolated metal atoms will be formed. Assisted with wet chemical or photochemical reduction, this strategy was used to synthesize SAECs with the frozen ice providing spatial confining environment. Wei and coworkers reported an ice-based photochemical reduction method to prepare Pt-based SAECs [31]. As illustrated in Figure 2(d), H_2_PtCl_6_ solution was rapidly frozen in liquid nitrogen followed by irradiation with UV lamp. After the H_2_PtCl_6_ ice was warmed to room temperature, a solution containing Pt single atoms was prepared. The obtained Pt single atoms in solution could be transferred onto multiwalled carbon nanotubes, mesoporous carbon, graphene, ZnO, and TiO_2_ by simple impregnation. This ice-based photochemical reduction method could also be used to prepare single-atom Ag- and Au-based catalysts. The most important factor to be precisely controlled is to guarantee the frozen state of the H_2_PtCl_6_ solution in the photoreduction process. Furthermore, this iced photoreduction method was extended to ice melting wet chemical reduction method with NaBH_4_ as the reducing reagent [32]. As shown in Figure 2(e), firstly, AgNO_3_ aqueous solution was rapidly frozen in liquid nitrogen. The ice was submerged into the NaBH_4_ solution and naturally melted with magnetic stirring at 0°C. After approximately one hour, the ice was partially melted, the remaining ice was removed. By collecting the mixed solution, the atomically dispersed Ag solution was prepared. The obtained single Ag atoms could also be supported onto mesoporous carbon. To be highlighted, this ice melting reduction method could be extended to fabricate other single-atom metal solutions including Co, Ni, Cu, Rh, Ru, Pd, Os, Ir, Pt, and Au. In a similar way, this liquid reduction method was used to prepare single-atom Co electrocatalyst (Co/NMC-LT900) at ultralow temperature of -60°C [47]. A CoCl_2_ solution (Solution A) and an alkaline N_2_H_5_OH and KOH solution (Solution B) were cooled down to -60°C, respectively. Then, Solution A was added dropwise into Solution B by a syringe pump. For the following reducing, anchoring, rinsing, and filtering process, the temperature was finely controlled at -60°C. After drying and annealing at 900°C for 1 h, single-atom Co/NMC-LT900 catalyst was prepared. Compared with traditional solution-phase reduction at room temperature, a sluggish nucleation rate and a higher energy barrier are required at ultralow reduction temperature to inhibit nuclei formation. As a result, single-atom cobalt catalysts are synthesized.

#### 2.1.6. Ion Exchange Method

With monodisperse Cu_1.94_S nanoparticles as the support, a redox-based ion exchange method was developed to prepare single-atom Pt catalysts (h-Pt_1_-CuS_*x*_) [48]. Firstly, H_2_PtCl_6_ solution was injected into a cyclohexane dispersion containing Cu_1.94_S nanoparticles. Then, Cu_1.94_S nanoparticles reacted with H_2_PtCl_6_. During the reaction, H_2_PtCl_6_ was reduced to Pt^0^ atoms, which were further embedded into the surface of the Cu_1.94_S nanoparticles to form single-atomic Pt sites in CuS_*x*_. The strong Pt-S interaction ensured that Pt atoms would selectively coordinate with S without the generation of Pt clusters. The interior of Cu_1.94_S was further oxidized and removed, finally yielding hollow CuS_*x*_ nanoparticles with rich isolated Pt sites (h-Pt_1_-CuS_*x*_). The Pt loading reaches as high as 24.8 at%. Kim et al. used a typical incipient wetness impregnation method to synthesize the atomically isolated Pt on antimony-doped tin oxide (ATO) [49]. Owing to the strong interaction of ATO with Pt, single-atom Pt was successfully anchored on ATO (Pt1/ATO) by substituting Sb sites with Pt atoms. The Pt loading is as high as 8 wt%.

### 2.2. High-Temperature Pyrolysis Methods

In 1964, Jasinski found that macrocyclic compounds with an MN_4_ center showed ORR activities [50], which opens up the avenue for preparation of SAECs by pyrolyzing C and N containing organic precursors (such as MOFs, melamine, dicyandiamide, ethylene diamine, polyaniline, phenanthroline, polypyrrole, phthalocyanine, porphyrin) together with transition metals (such as Fe, Co, Ni, Cu, Zn, Cr, Mn, Mo, Mg, Pt, Pd, Rh, Ir, Os) at high temperatures. Without chemical control, this high-temperature pyrolysis method usually causes the formation of metal nanoclusters/nanoparticles or metal oxides/carbides/nitrides, which makes the identification of the active-site structure a great challenge. In recent years, several effective strategies based on high-temperature pyrolysis have been revealed to synthesize SAECs with uniformly dispersed active sites.

#### 2.2.1. MOF-Based Encapsulation Pyrolysis

**Figure 3:**
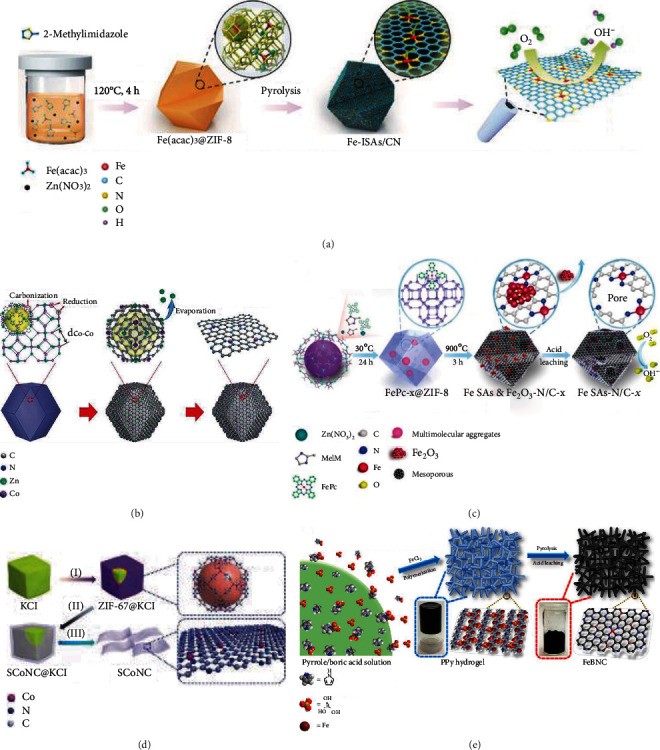
High-temperature pyrolysis methods for the synthesis of SAECs. (a) MOF encapsulation to synthesize Fe-based SAECs. Reproduced from [51]. (b) The synthesis of Co SAs/N-C. Reproduced from [52]. (c) The synthesis of single-atom Fe SAs-N/C-20. Reproduced from [53]. (d) The synthesis of the single-atom SCoNC catalysts with KCl molten salt method. Reproduced from [54]. (e) The synthetic procedure of single-atom FeBNC catalysts. Reproduced from [55].

To uniformly disperse active sites on atomic scale, metal organic frameworks (MOFs) are often used to synthesize the SAECs by pyrolysis at high temperatures. After heating treatment, the organic precursors were converted into N-doped porous carbon framework with isolated metal atoms. The catalytic activity of the resulting SAECs is of high sensitivity to the synthetic factors, such as the organic linker forms, the loadings of metal precursors, pyrolyzing atmosphere, and the temperature profiles. The utilization of MOFs together with a low content of metal precursors contributes to inhibit the migration and aggregation of metal species, resulting in the generation of dense single-atom metal sites. One of the most widely used MOFs is zeolitic imidazolate frameworks (ZIFs), especially ZIF-8. The nanocavity diameter of ZIF-8 is approximately 11.6 Å, which is large enough to encapsulate metal complexes such as Fe(acac)_3_ [51], Fe-Phen [56], and ferrocene (Fc) [57]. By enlarging the nanocavity size of ZIF-8, huge iron(II) phthalocyanine (FePc) molecules could be incorporated [53], as shown in Figure 3(c). After pyrolysis at 900°C under inert atmosphere, single-atom Fe-N-C catalysts were prepared. With FeCl_2_ and Zn(NO_3_)_2_ as the metal precursors, 2-methylimidazole as the organic ligand, and KI as the reducing reagent, Gu et al. prepared a Fe-doped ZIF-8 under nitrogen atmosphere [14]. After pyrolysis at 900°C, a single-atom Fe^3+^-N-C electrocatalyst was obtained. Compared with other methods conducted under ambient conditions, this approach under anaerobic conditions could synthesize a single-atom catalyst with Fe^3+^ ion center coordinated with pyrrolic N atoms of N-doped carbon support. It was found that the pyrrolic N ligands prefer to stabilize Fe^3+^, while the pyridinic N ligands are prone to coordinate with Fe^2+^. By ball-milling Fe(OAc)_2_, 1,10-phenanthroline, and ZIF-8, Li et al. prepared a FePhenMOF precursor [58]. Different from traditional one-step pyrolyzing under inert atmosphere, they proposed a two-step pyrolyzing approach: (i) Firstly, FePhenMOF was pyrolyzed under inert atmosphere (denoted as FePhenMOF-Ar). (ii) Then, FePhenMOF-Ar was further thermally treated under NH_3_ atmosphere (denoted as FePhenMOF-ArNH_3_). The second pyrolysis in NH_3_ maintains the active-site structure but remarkably elevating the density of available ORR sites by increasing the porosity directly contacting with the electrolyte and substrates. This MOF-based pyrolysis method could also be used to prepare binary metal SAECs. For instance, a binary metal Fe,Mn-N/C catalyst was prepared by adsorbing FeSO_4_ and manganese(II) 2,4-pentanedionate (C_10_H_14_MnO_4_) into the pores of ZIF-8 followed by pyrolyzing at 900°C [59]. The as-prepared Fe,Mn-N/C contains two types of M-N_*x*_ active site decorated in the carbon matrix.

The composition and morphology of SAECs could be modulated by coating ZIF-8 nanocrystals with customized layers. For example, by coating with PZS layers, an N, P, and S codoped Fe-SAs/NPS-HC catalyst was prepared [60]. In Fe-SAs/NPS-HC, the atomically dispersed Fe atoms coordinated with near-range nitrogen atoms and interacted with long-range sulfur and phosphorus atoms. Furthermore, with the silica coating layers, an overhang-eave structure anchored with isolated Fe atoms (Fe/OES) could be prepared [61]. Silica-coated MOFs could generate an outward adsorption force, which induces the MOF precursors toward anisotropic thermal shrinkage. As a result, the edge fringes of ZIF-8 were reserved, while the dodecahedron planar facets collapsed in the pyrolyzing process. Compared with traditional bulk carbon structure-supported single-atom Fe catalysts, the resulting Fe/OES catalyst showed edge-rich structure that processes much three-phase boundaries, which could enhance mass transport of substrates and improve the active-site accessibility. Surfactants were also used to coat MOF nanocrystals to prepare single-atom Co catalyst [62]. In comparison with cationic surfactant CTAB, anionic surfactant SDS, and nonionic surfactant PVP, nonionic triblock copolymer F127 showed strong interaction with Co-ZIF-8 nanocrytals by coordinating hydrophilic groups of F127 with Zn^2+^ and Co^2+^ sites on the surface of ZIF-8. During the subsequent pyrolysis, the F127 layers were firstly carbonized to form a carbon layer on the Co-ZIF-8 nanocrystals and showing significant confinement effect to inhibit the agglomeration of Co atoms. As a result, a single-atom Co catalyst was successfully prepared.

Zn cations in ZIF-8 could be gradually substituted with Co cations from 0 to 100 atom% with the ZIF structure unchanged. Yin et al. designed and prepared a Zn/Co bimetallic MOF with the molar ratio of Zn/Co above 1 : 1 [52]. By directly pyrolyzing the Zn/Co bimetallic MOF, a single-atom Co catalyst was obtained with Zn atoms evaporated away. As illustrated in Figure 3(b), the introduction of Zn atoms lengthened the distance between Co atoms and thus prevented the aggregation of Co species in the pyrolyzing process. The subsequent evaporation of Zn atoms could generate enriched porosity and high specific surface in the carbon matrix. With the Co doping content of 20 atom%, the obtained catalyst possessed the maximum density of isolated Co sites and exhibited the highest ORR activity [63].

While a large number of ZIF-8-derived SAECs have been prepared in recent years, high pyrolyzing temperatures above 900°C are usually needed to remove Zn from the carbon framework. The high-temperature treatment constantly leads to the formation of metal nanoclusters or nanoparticles. Suppressing the migration and aggregation of single-atom sites could preferentially increase the active-site popularity. The utilization of zinc limits the pyrolyzing conditions, especially pyrolyzing temperatures, in the optimization of ORR performance. Al-Zoubi and coworkers reported that by replacing Zn with a low-boiling-point Cd in the MOF, the pyrolyzing temperature could be dramatically dropped to 750°C [64]. In this protocol, firstly, with terephthalic acid and DABCO as the N-containing organic ligand, cadmium nitrate as the metal node, and iron nitrate as the Fe precursor, a binary-ligated Cd-Fe-DABCO-TPA MOF was prepared. Then, the as-made Cd-Fe-DABCO-TPA MOF and 1,10-phenanthroline were ball-milled for 2 h. After pyrolyzing at 750°C under NH_3_ atmosphere, a single-atom Fe-C-N_750_ was obtained. ICP-AES measurements demonstrated that most of Cd has been removed after the pyrolysis and the residual of Cd is only 0.4 wt% in the synthesized Fe-C-N_750_ catalyst. In contrast, 15.4 wt% of Zn was left in the ZIF-8-derived sample. The discrepancy in boiling point between Cd and Zn results in the residual amount of sacrificed metal in the produced catalyst.

With 2-amino terephthalic acid (H_2_BDC-NH_2_) as the organic linker, Wang and coworkers prepared an UiO-66-NH_2_ MOF [65], which contains lots of uncoordinated -NH_2_ groups. The strong interaction between Ru^3+^ and amine groups could guarantee the isolated Ru atoms to be confined in the pores of MOFs and prevent their aggregation in the pyrolyzing process. In the absence of amine groups, Ru atoms tend to aggregate with Ru clusters formed on the porous carbon. The same strategy could also be used to prepare single-atom W catalyst with WCl_5_ as the W precursor [66]. Liu et al. prepared an Mg-based MOF (Mg-HMT) by reacting MgCl_2_ with hexamethylenetetramine in a mixed solvent of water and alcohol [67]. After pyrolysis, the impurities were removed by diluted HCl solution. It should be mentioned that suitable drying conditions for the precursor play a decisive role in maintaining the high ORR activity. This is the first example to synthesize main-group-based SAECs by high-temperature pyrolysis.

#### 2.2.2. Molten Salt-Assisted Pyrolysis

Although SAECs derived from MOF precursors can preserve the original microstructures, this strategy also offers low yields and requires complex steps (especially the synthesis of precursors and post treatment). Therefore, for large-scale production and application of SAECs, it is a highly desirable task to propose simple and economical synthetic methods. Molten salts, including LiCl, NaCl, KCl, ZnCl_2_, and their mixtures, are soluble in water and could be easily removed after synthesis. As the template, molten salts could be used to synthesize nitrogen-doped porous carbon with a high specific area. More importantly, at high temperatures, the strong polarizing force of molten salts facilitates to break the ionic bonds in oxides and release free ions from solid oxides. After being trapped by N-doped carbon matrix, free ions were converted into isolated single-atom sites. In molten salts, the free migration of metal species could be inhibited, which is highly desired to form isolated metal sites at high temperature. For example, as shown in Figure 3(d), a KCl-assisted method was developed to prepare single-atom Co catalyst [54], in which a thin layer of ZIF-67 containing Co precursor grew on the surface of KCl particles, followed by annealing under argon at 750°C for 2 h and washing with diluted HCl and H_2_O to remove KCl template. With NaCl as the template, glucose as the carbon source, and FeCl_3_ as the Fe precursor, Zhang et al. prepared a single-atom Fe_1_-HNC-500-850 catalyst [68–70]. By mixing NaCl, glucose, and FeCl_3_ in water followed by freeze drying, grinding, and pyrolyzing under NH_3_ at 500°C and Ar at 850°C, single-atom FeN_4_ sites were anchored on honeycomb-like nanosheets (Fe_1_-HNC-500-850). Similarly, Ni_1_-HNC-500-850 and Co_1_-HNC-500-850 catalysts could also be prepared with this NaCl-assisted strategy.

The molten salt-assisted strategy makes it possible to utilize ordinary metal oxides (such as Fe_2_O_3_ and Co_2_O_3_) and small organic molecules (such as adenine) as precursors to produce carbon-supported SAECs [71]. Firstly, the molten salt (ZnCl_2_ and NaCl with a mass ratio of 1 : 1), adenine, and Fe_2_O_3_ were ground by ball mill. The obtained sample was thermally treated at 900°C under inert atmosphere. After removal of the impurities, the single-atom Fe-SAC/NC catalyst was obtained. The strong polarizing force of molten salts plays critical roles in dissolving oxides, releasing free metal ions, and forming N-doped carbon framework to trap generated metal ions. In addition, the molten salt could also modulate the structure of the carbon framework; in that, ZnCl_2_ with a boiling point of 732°C will be evaporated at high temperatures above 800°C and can act as a suitable pore-forming agent. In contrast to traditional thermal treatment, the pyrolysis of small organic molecules in molten salts could suppress the direct decomposition of these molecules and promote the carbonization process.

#### 2.2.3. Polymer Pyrolysis

Polymers are a kind of macromolecules and consist of repeated subunits. By selecting suitable subunits, coordination atoms including N, S, and P could be incorporated into polymers. Corresponding polymers could be used as the precursors of carbon matrix and doped heteroatoms. Various synthetic or natural polymers, such as polypyrrole [55], pyrrole-thiophene copolymer [72], poly(2-fluoroaniline) [73], chitosan [74], polydopamine [75], and cocoon silk [76], have been successfully used to synthesize SAECs. For example, with pyrrole, phytic acid, and FeCl_3_ as the precursor, Yuan et al. prepared a binary nitrogen and phosphorus-coordinated single-atom iron catalyst (Fe-N/P-C) [77]. Firstly, polypyrrole (PPy) hydrogel was synthesized by the polymerization of pyrrole with phytic acid and FeCl_3_. Then, the PPy hydrogel was thermally treated under inert atmosphere. After leaching with diluted sulfuric acid, nitrogen and phosphorus binary-coordinated iron active sites decorated on carbon nanosheets were prepared. As shown in Figure 3(e), by replacing phytic acid with boric acid, this pyrolysis method based on polypyrrole (PPy) hydrogel could also be used to prepare B-doped Fe-N-C catalyst (FeBNC) containing boron centers and atomically dispersed Fe-N_*x*_ sites [55].

With polypyrrole and FeCl_3_-coated carbonaceous nanofibers (CNF@PPy) as the precursor and SiO_2_ as the protecting layer, a single-atom Fe-N-doped carbon nanofiber catalyst (Fe-N-CNF) was synthesized [78]. In the absence of the SiO_2_-protected shell, Fe species tends to aggregate to form inactive nanoparticles in the pyrolyzing course. The silica-protected shell plays important roles in suppressing the aggregation of iron species and trapping volatile chemicals in the pyrolysis process. As a result, the obtained Fe-N-CNF catalysts showed optimized surface functionalities and porous structures. By coating poly(2-fluoroaniline) (P2FANI) onto FeO-(OH) nanorods followed by controlled pyrolysis at high temperatures, Zhang et al. synthesized a mesoporous Fe-N/C catalysts. The FeO-(OH) nanorods serve as both the hard template to suppress the shrinkage of P2FANI in the carbonization process and the pore-forming agent to increase the porosity in the carbonized framework. The obtained Fe-N/C possesses a specific surface area as high as 934.8 m^2^ g^−1^.

By introducing metal precursors during the in situ condensation process of formamide, atomically dispersed dense metal sites coordinated with multiple N-dentate ligands formed. After pyrolysis, SAECs could be obtained [79]. This approach could produce seven kinds of monometallic (Fe, Co, Ni, Mn, Zn, Mo, and Ir), one kind of bimetallic (FeCo), and one kind of trimetallic (ZnFeCo) SAECs. In addition, all of the resulting SAECs could be transferred onto various substrates (graphene oxide, CNTs, active carbon, and silica) as ultrathin layers. Among the prepared catalysts, the pyrolysis-free Fe and Ni single-atom electrocatalysts supported onto CNTs exhibited excellent ORR performance.

Very recently, controlled shockwaves with high temperatures were introduced to synthesize single-atom catalysts. This technique operates at very high temperatures (1500-2000 K) and features with a periodic on-off heating process [80]. Firstly, small amounts of metal salts were dissolved in ethanol to form a diluted solution (0.1-0.5 mmol L^−1^). The resultant solution was transferred onto the carbon nanofiber (CNF) film with a loading of 20-100 *μ*L cm^−2^ (in total ~0.01 *μ*mol cm^−2^). The precursor-loaded CNF films were then treated with a programmed electrical pulse under inert atmosphere. The on-state high temperature offers energy to overcome the activation barrier for atom dispersion and form thermodynamically favorable metal-defect bonds, while the off-state guarantees the stabilization, especially for the substrate. The as-prepared single-atom catalysts show superior thermal stability and could be used as durable catalysts. This shockwave method could be used to prepare Pt, Ru, and Co single atoms on various substrates including carbon, C_3_N_4_, and TiO_2_. In a similar way, Bi and coworkers developed an arc-discharge method to prepare single-atom Pt_1_/MoC catalyst. The high temperature of up to 4000°C overcomes the energy barrier for Pt atom dispersion and overall stability by forming thermodynamically favorable metal-support interactions, which enables the Pt_1_/MoC catalyst exhibiting ultrahigh thermal stability [81].

#### 2.2.4. Metal Organic Complexes Pyrolysis

Metal organic complexes contain metal atoms and organic ligands simultaneously. Different organic ligands contain different coordination atoms, show different molecular sizes, and exhibit different decomposition temperatures. Commonly used organic ligands include porphyrins, phthalocyanines (Pc), 1,10-phenanthroline (Phen), bipyridine, and acetylacetone (acac). To be highlighted, some metal organic complexes such as metal (iron, cobalt, and manganese) porphyrins and phthalocyanines could be directly used as the electrocatalysts for ORR [50]. In most cases, metal organic complexes serve as the precursors of metal, C, and other heteroatoms to synthesize SAECs by pyrolysis. With Fe-porphyrin complexes as the metal precursor and carbon nanotubes (CNT) as the support, Sa et al. developed a general “silica-protective-layer-assisted” method that can preferentially generate the catalytically FeN_4_ sites doped in CNT walls while inhibiting the formation of inactive Fe-based particles [82]. Firstly, iron porphyrin precursors were adsorbed onto CNTs followed by silica layer overcoating and high-temperature pyrolysis. After removal of silica layer by acid etching, thin layer porphyrinic carbon coating on CNT catalysts (CNT/PC) was prepared. Temperature-controlled in situ X-ray absorption spectroscopy in the synthetic process of CNT/PC reveals that the interaction between the silica protecting layers and the FeN_4_ moieties prevents to form large Fe-based nanoparticles. Compared with the CNT/PC prepared without silica protective layers, the CNT/PC catalyst contains higher density of FeN_4_ sites. This silica-protected-layer-assisted approach could be extended to prepare other carbon-supported SAECs including Ketjen black, reduced graphene oxides, acetylene black, and Vulcan. By diluting metalloporphyrin (MTPP) containing target metals with tetraphenylporphyrin (TPP) diluents followed by pyrolysis, twenty-four kinds of single-atom electrocatalysts including noble and nonnoble metals (such as Pt, Pd, Ru, Rh, Au, Ag, Ir, Ti, V, Cr, Mn, Fe, Co, Ni, Cu, Ga, Zr, Mo, W, Cd, In, Sn, Er, Bi) could be prepared [83, 84]. It should be noted that this high-temperature pyrolysis method requires expensive equipment and consumes high energy, which must be considered for commercial applications.

### 2.3. Vapor Deposition Method

#### 2.3.1. Atomic Layer Deposition (ALD)

The atomic layer deposition (ALD) technology is based on cyclically sequential self-terminating reactions between gas-phase precursor molecules and a solid surface. In a typical ALD process, the precursor molecules are introduced to contact with the solid surface and then react with specific sites on the solid surface. In case all reactive sites have been consumed, the growth process stops. The self-limiting feature of ALD provides controllable and accurate means to synthesize SAECs.

**Figure 4:**
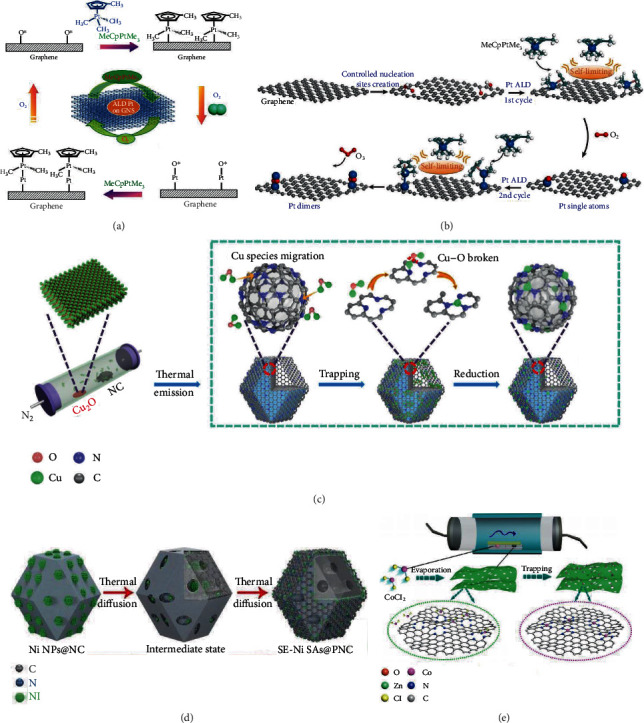
Vapor deposition and atom-trapping methods for the synthesis of SAECs. (a) Synthesis of atomically isolated Pt on graphene nanosheets with the ALD method. Reproduced from [85]. (b) Preparation of Pt_2_/graphene catalysts. Reproduced from [86]. (c) Synthesis of single-atom Cu catalyst with the atom trapping method. Reproduced from [87]. (d) Synthesis of single-atom Ni catalyst from Ni NPs. Reproduced from [88]. (e) Synthesis of single-atom Co catalyst. Reproduced from [89].

A single-atom Pt/Graphene catalyst was prepared through the atomic layer deposition (ALD) method with (methylcyclopentadienyl)-trimethyl platinum (MeCpPtMe_3_) and oxygen molecules as the precursors [85]. As shown in Figure 4(a), MeCpPtMe_3_ firstly reacted with oxygen atoms of the graphene sheets to form Pt-containing monolayer, which further reacted with oxygen molecules to prepare single-atom Pt sites coordinated with oxygen atoms. The popularity of Pt atoms on graphene substrate can be precisely tuned by controlling the cycle numbers. For instance, after 50 ALD cycles, the Pt loading was as high as 1.52 wt% and the deposited Pt species consisted of isolated Pt atoms as well as small fraction of Pt nanoclusters. With graphene oxide sheets replaced by nitrogen-doped graphene nanosheets (NGNs), this ALD method could be used to prepare single-atom Pt/NGNs catalysts [90]. In a similar way, palladium hexafluoroacetylacetate (Pd(hfac)_2_) and bis(cyclopentadienyl) cobalt (Co(Cp)_2_) could also be used as the metal precursors to prepare corresponding SAECs with the ALD technique [91, 92]. Yan et al. further found that Pt_2_ dimers could also be deposited on graphene with ALD technology [86]. As shown in Figure 4(b), phenol-related oxygen species on graphene function as anchor sites to immobilize Pt and the Pt_2_ dimers are likely in the oxidized Pt_2_O_*x*_ form. It must be mentioned that expensive apparatus and low yields limit the application of this ALD method on a large scale.

#### 2.3.2. Chemical Vapor Deposition (CVD)

Chemical vapor deposition (CVD) techniques have been widely utilized to produce two-dimensional materials [93, 94]. However, the application of this CVD technique to synthesize SAECs has remained less popular. Qiu et al., for the first time, developed a CVD method to embed isolated Ni atoms in graphene nanosheets [95]. By treating Ni_30_Mn_70_ alloy with (NH_4_)_2_SO_4_ aqueous solution, they prepared nanoporous Ni substrates, followed by a CVD process to coat graphene layers. After removal of the Ni substrate with 2.0 M HCl solution, single-atom Ni decorated in porous graphene nanosheets was obtained. The Ni atoms are physically adsorbed on the hollow centers of graphene. Furthermore, by replacing benzene with pyridine, they synthesized single Ni atoms/clusters immobilized in N-doped nanoporous graphene using this CVD process [96], in which N atoms were firstly predoped followed by Ni deposition. In the resultant catalyst, the total Ni loading was up to 23 wt%, including isolated atoms and clusters. The atomically dispersed Ni atoms were stabilized by both Ni-N bonds and Ni-C bonds.

### 2.4. Atom Trapping Method

Atom trapping method is a newly developed strategy to synthesize SAECs. At high temperatures, metal nanoparticles (Pt, Pd, Au, Rh, and Ni nanoparticles) [88, 97, 98], metal bulks (Pt net, Au foil, Pd bulk, Nb bulk, Cu foam, Co foam, and Ni foam) [99–101], metal oxides (Fe_2_O_3_, Co_2_O_3_, Cu_2_O, SnO_2_, and MoO_3_) [87], and metal salts (CoCl_2_, H_2_PtCl_6_, H_2_PdCl_4_, HAuCl_4_, and H_2_IrCl_6_) [89, 102] could release metal atom vapor, which could be trapped by various substrates placed in the downstream and form corresponding SAECs. For example, Nb atom vapor released by arc discharge of Nb bulk could be trapped by carbon and formed the single-atom Nb-in-C catalyst [99]. As shown in Figure 4(c), under nitrogen atmosphere, the surface of commercial Cu_2_O could be evaporated to form volatile species at 1273 K and the volatile species could be trapped and reduced by N-doped carbon, N-doped reduced graphene oxide, and N-doped carbon nanotubes to generate single-atom Cu-based catalysts [87]. In a similar way, Sn-based and Mo-based single-atom catalysts were also prepared by using SnO_2_ and MoO_3_ as the metal precursors. This approach avoids the usage of corrosive NH_3_ and is suitable for the large-scale production and practical application. Wei and coworkers directly observed the transformation of Pt, Pd, and Au nanoparticles into their corresponding single atoms in the presence of N-doped carbon above 900°C in an inert atmosphere by in situ environmental TEM [98]. DFT calculations suggested that the high-temperature conversion of noble metal nanoparticles to N-coordinated single-atom catalysts was driven by the formation of more thermal dynamically stable M-N_4_ (M=Pt, Pd, and Au), while migrating noble metal atoms were trapped by the defect sites on N-doped carbon substrates. They also found that besides nanoparticles, bulk metals (such as Cu, Co, and Ni foam) could react with NH_3_ at high temperatures to form Cu(NH_3_)_*X*_, Co(NH_3_)_*X*_, and Ni(NH_3_)_*X*_ species [100]. After trapped by N-doped carbon framework originated from ZIF-8 by pyrolysis, single-atom catalysts were obtained. To be highlighted, this strategy is suitable to practical applications.

Feng et al. reported a general strategy to disperse active carbon-supported Rh nanoparticles into single-atom Rh catalyst [103]. Firstly, active carbon-supported RhCl_3_ was thermally treated under nitrogen atmosphere at 300°C for 2 h. The residual was reduced with hydrogen at 300°C for 2 h to obtain the fresh 5 wt% Rh catalyst with Rh nanoparticle size of 4 nm (RhNP/AC). The RhNP/AC was transformed into the single-atom Rh_1_/AC catalyst by heating at 240°C for 6 h in a CO/CH_3_I mixture. It was found that CO molecules and in situ generated I radicals promoted the breakage of Rh-Rh bonds to form mononuclear Rh(CO)_3_I complexes, which were trapped by oxygen-containing active carbon to form the single-atom Rh(CO)_3_I(O-AC) catalyst. This approach could also be used to transform active carbon-supported Ru, Pd, Ag, Ir, and Pt nanoparticles to corresponding single-atom catalysts.

Besides evaporation at high temperatures, synthesis of SAECs with the atom trapping method at low temperatures is also realized. Zhang and coworkers developed a synergistic micropore trapping and nitrogen anchoring method to prepare Pt single-atom catalysts (Pt_1_/hNCNC) [102]. Both DFT calculations and experiments suggest that the micropores decorated with nitrogen atoms (especially pyridinic N) on the edge are favorable for the dispersion of platinum atoms; in that, H_2_PtCl_6_ could react with amino group to form stable [C_*x*_(NH)_2_]^2+^[PtCl_6_]^2-^ via the electrostatic interaction, followed by the spontaneous dechlorination upon heat treatment at 70°C. This method could also be successfully applied to construct Pd-, Au-, and Ir-based SAECs.

### 2.5. General Synthetic Methods for SAECs with High Metal Loadings on Practical Scale

SAECs exhibit tremendous potential in electrocatalysis. However, the scalable synthesis of SAECs with high density of active sites is of great challenge, due to the balance between single-atomic dispersion and loading in the formation of M-N-C sites at high temperatures. For practical applications, the synthesis of SAECs on a large scale with generality is highly required. In very recent years, great progress has been made [104]. For example, the loading of isolated metal atoms has been as high as 18 wt% [105], the general method has been extended to synthesize SAECs for more than 34 types [102], and the production of SAECs on a kilogram scale has been realized [106]. Finally, the introduction of the deep learning algorithm together with big data technology will greatly speed up the screening process and start up a new direction of rational design and modification for complicated SAECs with expected electrochemical catalytic performance.

**Figure 5:**
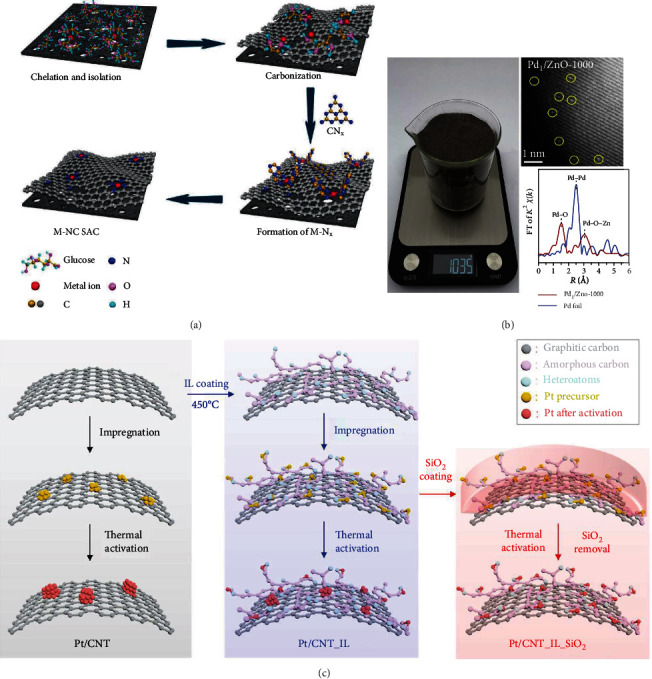
General synthetic methods for the synthesis of SAECs on practical scale. (a) The preparation of M-NC SACs with the cascade anchoring strategy. Reproduced from [107]. (b) Preparation of Pd1/ZnO on kilogram scale. Reproduced from [106]. (c) Synthesis of single-atom Pt/CNT_IL_SiO_2_. Reproduced from [108].

As shown in Figure 5(a), a cascade anchoring strategy was developed for the mass production of a series of M-N-C SAECs (M=Mn, Fe, Co, Ni, Cu, Mo, Pt) with a metal loading up to 12.1 wt% [107]. Firstly, glucose molecules chelate with metal ions and bind to O-rich carbon support. Excessive glucoses were used to isolate glucose-metal complexes on the carbon substrate. Then, the chelated metal complexes release metal atoms in the pyrolysis process at high temperatures, which were captured by decomposed CN_*x*_ species from melamine to generate M-N_*x*_ moieties and embed into the carbon matrix to form SAECs. By reacting Ir(CO)_2_(acac) with O-containing groups on the reduced graphene aerogel (rGA), atomically isolated iridium complexes could be immobilized on rGA. The rGA substrate provides highly effective bonding sites for metal anchoring superior to those of metal oxides, due to the merits of uniformity and high density. This approach could prepare a single-atom Ir catalyst with remarkably high Ir loading up to 14.8 wt% [109]. With carbon cloth- (CC-) supported NiO (NiO/CC) as the support, Wang et al. prepared a single-atom Ir catalysts with the Ir loading as high as 18 wt% [105]. Firstly, a piece of NiO/CC was immersed into a chloroiridic acid ethanol solution for 10 min and then dried at 80°C. Subsequently, the dried sample was calcined at 350°C for 2 h in air, cooled down, and washed with water. The Ir-NiO/CC catalyst was obtained after dried in air. It was observed that atomically dispersed Ir-atoms are anchored at the outermost surface of NiO and are stabilized by covalent Ir-O bonding, which induces the isolated Ir atoms to form a favorable Ir(IV) oxidation state.

A general precursor dilution strategy was developed to prepare SAECs. Firstly, metalloporphyrin (MTPP) containing target metals copolymerized with tetraphenylporphyrin (TPP). The TPP molecules were used as diluents to separate metal atoms. After pyrolysis at high temperatures, carbon-supported single-atom catalysts (M_1_/N-C) were obtained [83]. By using this method, twenty-four kinds of single-atom catalysts including noble and nonnoble metals (such as Pt, Pd, Ru, Rh, Au, Ag, Ir, Ti, V, Cr, Mn, Fe, Co, Ni, Cu, Ga, Zr, Mo, W, Cd, In, Sn, Er, and Bi) were successfully prepared. This method could also be extended to prepare bimetallic Pt_1_-Sn_1_/N-C single-atom catalysts. Furthermore, a general electrochemical deposition strategy applicable to a wide range of metals and supports was developed to prepare SAECs. In a standard three-electrode device, a glassy carbon electrode loaded with Co(OH)_2_ nanosheets were used as the working electrode and a diluted H_2_IrCl_6_ solution was used as the metal precursor and the electrolyte. The depositing process started from 0.10 to -0.40 V in cathodic deposition and from 1.10 to 1.80 V in anodic deposition. The scanning cycle was repeated for three times to obtain A-Ir_1_/Co(OH)_2_ from the anode and ten times to obtain C-Ir_1_/Co(OH)_2_ from the cathode. More than 30 kinds of different SAECs (Fe, Ni, Co, Zn, Cu, Cr, V, Ag, Mn, Ru, Ir, Pd, Rh, Pt, Au, etc.) are prepared from anodic or cathodic deposition by changing supports and metal precursors [46].

With carbon black as the support, 1,10-phenanthroline (Phen) as the N-containing ligand, and transition metal salts as the metal precursor, a general ligand-mediated strategy was developed to prepare transition metal-based SAECs on a large scale [110]. Firstly, nickel(II) acetate coordinated with 1,10-phenanthroline in ethanol to form Ni-Phen complexes. Then, Ni-Phen complexes were adsorbed onto carbon black support. After pyrolysis under argon atmosphere, single-atom Ni-SAC catalyst was obtained. The Ni loading could be as high as 5.3 wt%. By changing the metal precursors, Mn-, Fe-, Co-, Zn-, Cr-, Cu-, Ru-, and Pt-based SAECs and Fe/Co-, Ru/Fe-, Ru/Co-, and Ru/Ni-based binary SAECs were also successfully prepared. To be highlighted, this synthetic approach could be enlarged to produce carbon-based SAECs on a kilogram scale. By ball-milling iron(II) acetate, 1,10-phenanthroline (Phen), and ZIF-8, Sun et al. prepared a FePhenMOF precursor [39]. After thermal treatment of FePhenMOF under Ar and NH_3_ atmosphere, a single-atom FePhenMOF-ArNH_3_ could be prepared. This ball-milling method could be easily scaled up to synthesize gram-level SAECs in one pot depending on the size of furnace and ball-milling machine. Furthermore, this method was extended to prepare ZnO- and CuO-supported Pd-based single-atom catalysts on a kilogram scale [106]. As shown in Figure 5(b), firstly, the mixture of Pd(acac)_2_ and Zn(acac)_2_ with a weight ratio of 1 : 400 was thoroughly grounded. After calcination at 400°C for 2 h in air, the single-atom Pd_1_/ZnO catalyst was obtained. With the same method, ZnO-supported Rh- and Ru-based single-atom catalysts were also prepared on a kilogram scale.

With the high-speed development of artificial intelligence, the deep learning algorithm attracted more and more attention in a broad research field. Sun et al. for the first time used the deep learning algorithm to develop graphdiyne-supported SAECs with zero-valenced central metal atoms [111]. By quantifying the electron transfer ability and zero-valence stability between metals and graphdiyne support, it was found that among all transition metals, Co, Pd, and Pt showed exceptional stability of zero-valence SAECs based on the evident energy barrier difference between losing electrons and gaining electrons. This novel deep learning algorithm together with big data technology starts up a new direction of rational design and modification for complicated SAECs with expected electrochemical catalytic performance.

## 3. Characterizations

Compared with nanoclusters and nanoparticles, single-atom electrocatalysts possess well-defied ORR active sites, which simplifies the model construction for theoretical calculations and improves the deeply understanding of the interaction between active sites and substrate species. Conversely, the understanding of fundamental reaction mechanism further directs the design of more highly efficient ORR catalysts by tuning the geometric configurations and coordination environments at the atomic level. Experimentally, the characterization and identification of single-atom sites can be completed with advanced techniques including synchrotron-based X-ray absorption spectroscopy, Mossbauer spectroscopy, scanning tunneling microscopy, and scanning transmission electron microscopy. The atomic-level structure information, including the chemical state, the coordination environment, the spin state of metal centers, and electronic properties, could be elucidated. The combination of these advanced characterization techniques with DFT calculations can clarify the structure-performance relationship, enabling precise rational design of customized SAECs. The characterization techniques for SAECs in this review include scanning tunneling microscopy (STM), sub-angstrom-resolution aberration-corrected scanning transmission electron microscopy (AC-STEM) equipped with EDX and EELS accessories, synchrotron-based X-ray absorption spectroscopy (XAS) including XANES and EXAFS, Mössbauer spectroscopy, X-ray diffraction (XRD), X-ray photoelectron spectroscopy (XPS), Fourier-transform infrared spectroscopy (FTIR), nuclear magnetic resonance (NMR), inductively coupled plasma atomic emission spectrometry (ICP-AES), elemental analysis, and density functional theory (DFT) calculation. ORR performance of SAECs is usually tested on rotating disc electrode (RDE) or rotating ring disc electrode (RRDE) in various oxygen-saturated electrolytes, such as 0.1 M alkaline KOH solution, 0.05 M neutral phosphate-buffered solution (PBS), 0.5 M acidic H_2_SO_4_ solution, and 0.1 M acidic HClO_4_ solution.

### 3.1. X-Ray Diffraction

X-ray diffraction (XRD) is the most commonly used indirect characterization technology to prejudge the formation of SAECs [30, 51, 56]. The principle is based on the following: (i) If diffraction peaks ascribed to the metal-related species (including metal, metal oxides, metal carbides, metal nitrides, metal sulfides, metal phosphides) are detected, the obtained catalysts could not be called single-atom electrocatalysts. (ii) If the single-atom catalysts are successfully synthesized, no diffraction peaks ascribed to the metal-related species mentioned above could be detected. But this prejudgment is insufficient, because no diffraction peaks might be attributed to the low metal loading (<2 wt%) or the very small particle size (<4 nm). Some other advanced characterization technologies should be further conducted.

### 3.2. Scanning Tunneling Microscopy

**Figure 6:**
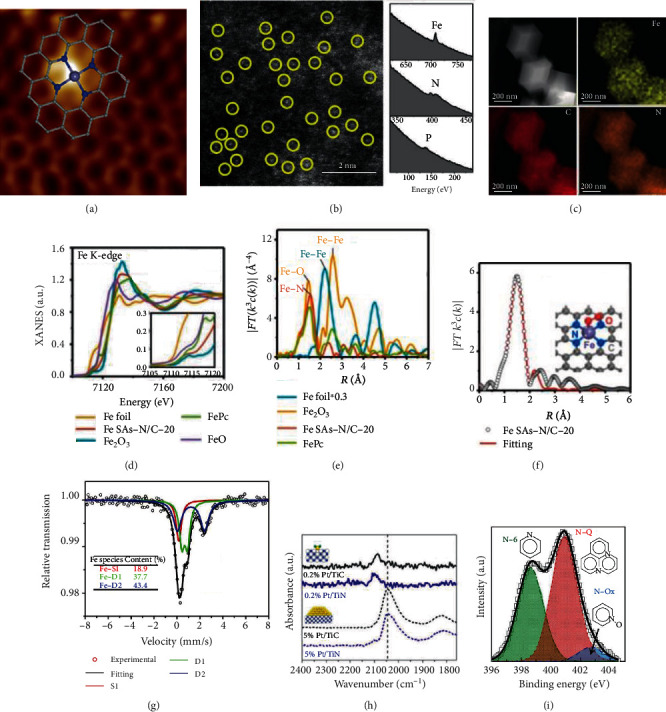
Characterization techniques for SAECs. (a) STM image of single-atom FeN_4_/GN-2.7 (2 nm × 2 nm). Reproduced from [112]. (b) AC-STEM image of Fe-N/P-C-700 and corresponding EELS atomic spectra of Fe, N, and P elements from the bright dots shown in the yellow circle. Reproduced from [77]. (c) HAADF-STEM image and element mapping of single-atom Fe catalyst. Reproduced from [51]. (d) XANES spectra of Fe K-edge. Reproduced from [53]. (e) FT *k*^3^-weighted EXAFS spectra. Reproduced from [53]. (f) Fitting curves of FT-EXAFS. Reproduced from [53]. (g) Mössbauer spectrum of ^57^Fe. Reproduced from [77]. (h) Diffuse reflectance FT-IR spectra of adsorbed CO. Reproduced from [113]. (i) XPS spectra of N 1 s. Reproduced from [114].

Scanning tunneling microscopy (STM) is an ultrahigh-resolution instrument and commonly used to observe the surface structure at the atomic level. With a conducting tip approaching to the substrate surface, a bias voltage will be exerted. Meanwhile, the generation of a tunneling current occurs. By recording the current as the tip scanning across the surface, the structure information of the substrate surface can be detected. As shown in Figure 6(a), in a low-temperature STM image of N-doped graphene-based single-atom Fe catalyst, the isolated Fe site was observed, which resolved as a brighter spot and coordinated with four nitrogen atoms embedded in the graphene matrix [112]. Patera and coworkers used an in situ high-speed STM to directly monitor the catalytic behavior of single Ni atoms at the Klein (k) and Zigzag (z) edges of a graphene flake during real growth process [115]. They found that Ni atoms reside longer in the kink sites of graphene accompanied by C dimer attachment nearly, which suggests that single Ni atoms play the catalytic roles for the graphene growth.

### 3.3. Aberration-Corrected Scanning Transmission Electron Microscopy

In recent years, the resolution of an aberration-corrected scanning transmission electron microscopy (AC-STEM) has been increased to sub-angstrom of 0.5 Å, which makes it capable to distinguish isolated single atoms. AC-STEM image provides the most effective method to directly observe isolated metal atoms anchored on the supports based on the Z-contrast imaging. By focusing electron beam on the sample and collecting electrons that scatter out to an annular dark field detector, Z-contrast images can be obtained, where Z represents the atomic number of the observed atom. The electron beam energy might induce the dynamic hopping and movement of isolated atoms, which leads to inaccurate atomic images. When we carry out AC-STEM experiments, we should guarantee that the tested samples could tolerate to the electron beam and take measures to mitigate the negative effect of electron beam. Accompanied by energy-dispersive X-ray spectrometry (EDX) and electron energy loss spectroscopy (EELS), element composition and distribution at the atomic level can be obtained [14, 90]. In 1996, Nellist and Pennycook for the first time resolved the position of the isolated metal atom from substrates by using the Z-contrast atomic resolution imaging [116]. By means of in situ environmental transmission electron microscopy, Wei and coworkers directly observed the process of converting Pd nanoparticles into isolated Pd atoms at high temperatures in the presence of N-doped carbon support [98].

### 3.4. X-Ray Absorption Spectroscopy

Electron microscopy enables the direct observation of isolated single atoms. However, the visual range with this technology is limited and only specific local area can be selected. Synchrotron-radiated X-ray absorption spectroscopy (XAS) is a more powerful technique to confirm the overall detailed atomic and electronic information of selected single atoms. XAS relies on the oscillatory structure in the X-ray absorption coefficient above an X-ray absorption edge. These signals precisely reflect the structure information of the materials, including the oxidation state, the coordination number, and the coordination environments. Therefore, XAS is an effective technology to confirm the chemical state and the local atomic structure of isolated atoms. Generally, X-ray absorption near-edge spectroscopy (XANES) and extended X-ray absorption fine structure (EXAFS) are two typical measurement regimes of XAS. XANES measures the oxidation state and coordination environment (e.g., tetrahedral, hexahedral, octahedral coordination) of the selected atoms, while EXAFS provides information on the distance, coordination number, and species of the neighbor elements of the absorbing atoms. With XANES and EXAFS, the structures of series of SAECs, such as FeN_4_, CoN_4_, CoN_2+2_, NiN_2_O_2_, MoN_1_C_2_, WN_4_, WN_1_C_3_, and WN_2_C_2_, have been successfully confirmed [53, 62, 66, 74, 117]. In addition, the structures of CoN_2_, CoN_3_, CoN_4_, and CoN_5_ have also been synthesized and characterized [33, 118]. With *in situ* XAS techniques, Li et al. identified the Fe^2+^-N_4_ moieties with nonplanar configuration embedded in carbon matrix as the highly efficient ORR active sites [58]. This nonplanar Fe^2+^-N_4_ configuration could reversibly transform to an in-plane Fe^3+^-N_4_ moiety axially coordinated with oxygen adsorbates when the applied potential crosses the Fe^2+/3+^ redox potential anodically. The density of the active sites could be modulated by the redox potential. With the assistance of in situ XAS technologies, Sasaki et al. observed the deactivation mechanism of Pt nanoparticle catalysts during the ORR process, which follows the oxidation of the outermost Pt atoms and then the dissolution caused by carbon corrosion and the OER process [119]. Kaito et al. revealed that the Pt-Pt bond length dominates the intrinsic ORR activity [120]. Yoshida et al. confirmed that the oxygen evolution reaction occurs at the domain edge of NiO_6_ octahedra of the nickel borate electrocatalyst [121]. These findings are totally different from previously reported results based on *ex situ* techniques.

### 3.5. Mössbauer Spectroscopy

Mössbauer spectroscopy is a Mössbauer effect-based characterization technique. Mössbauer spectroscopy is composed of the nearly recoil-free emission and absorption of nuclear gamma rays in solids. Three typical nuclear interactions may be detected: the isomer shift generated by differences in nearby electron densities, quadrupole splitting produced by atomic-scale electric field gradients, and magnetic Zeeman splitting created by nonnuclear magnetic fields. Mössbauer spectroscopy featuring with high energy and narrow line widths of nuclear gamma rays is a highly sensitive technique to detect the energy changes in 10^11^ resolution. Based on ^57^Fe-based Mössbauer technique, Fe-based SAECs have been precisely characterized owing to their ultrasensitivity to the coordination environment and electronic configuration of the central Fe ions in the complicated materials [58].

Mössbauer spectroscopy is a more powerful technique to detect the purity of Fe-based SAECs. For instance, Kramm et al. fount that Fe-based SAECs prepared by pyrolyzing Fe-porphyrin complexes at 800°C showed three doublets (D1, D2, and D3) and a singlet [122]. Three doublets could be attributed to FeN_4_ sites with a ferrous low-spin (D1) and to two ferrous mid-spin (D2 and D3) sites. D1 with in-plain low-spin (S = 0) ferrous FeN_4_ sites is regarded as the active center for 4e^−^ ORR in acidic electrolytes. The difference between D2 sites and D3 sites lies in their local environments, i.e., D2 resembling iron phthalocyanine (FePc) and D3 similar to porphyrins. D2 offers negligible contribution to the ORR activity due to the axial coordination with adjacent N atoms of FePc molecules, which inhibits the adsorption and activation of oxygen molecules. D3 is stabilized by carbon frameworks. The singlet is belonging to superparamagnetic iron, i.e., very small iron nanoparticles without magnetic ordering. The appearance of the singlet indicates the formation of impurities in Fe-based SAECs. After purification by conducting a second thermal treatment of the resultant catalysts in the forming gas with a subsequent HCl leaching, the singlet disappeared and only doublets remained in the Mössbauer spectroscopy, demonstrating the formation of single-atom FeN_4_ electrocatalysts with high purity.

Mössbauer spectroscopy could also be used to analyze the structure of Fe-based binary metal SAECs and binary ligand-coordinated Fe-based SAECs. Wang et al. prepared a Fe-based single-atom catalyst (Fe SAs/N-C) and a Fe-Co binary metal single-atom catalyst ((Fe,Co)/N-C) [123]. The Mossbauer spectrum of Fe SAs/N-C is composed of three doublets (D1, D2, and D3) that could be attributed to square-planar Fe(II)N_4_ configuration with Fe(II) in a high-, low-, and intermediate-spin state, respectively, while the Mossbauer spectrum of (Fe,Co)/N-C could be fitted with three FeN_4_ centers similar to those of the Fe SAs/N-C and a singlet. The singlet demonstrates the presence of Fe-Co bond in (Fe,Co)/N-C.

Yuan et al. prepared binary N and P-coordinated single-atom Fe catalysts (Fe-N/P-C-700) with FeN_3_P configuration as the active site [77]. As shown in Figure 6(g), the Mossbauer spectrum of Fe-N/P-C-700 was composed of one singlet assigned to Fe-P and two doublets (D1, D2) ascribed to Fe-Nx moieties. The D1 and D2 doublets are belonging to square planar Fe-N_4_ configurations. In the Mössbauer spectrum, no sextets were detected, indicating the absence of iron carbides in Fe-N/P-C-700 and all Fe species are atomically dispersed.

### 3.6. Fourier-Transform Infrared Spectroscopy

One of the most important features of single-atom catalysts is the absence of nanoclusters and nanoparticles. AC-STEM could directly observe the dispersion of single atoms. However, the existence of nanoclusters and nanoparticles could not be excluded. Fourier-transform infrared spectroscopy (FTIR) can resolve and record the vibration mode of adsorbed probe molecules on catalyst surfaces, which could be used to distinguish the differences between single-atom sites and nanoparticle sites [5, 124]. With CO as the probe molecules, in situ FTIR provides a powerful tool to identify the absence of nanoclusters and nanoparticles by monitoring the highly sensitive vibration mode of the adsorbed CO molecules. As shown in Figure 6(h), for single-atom Pt catalysts, such as Pt_1_/FeO_*x*_, Pt_1_/TiC, and Pt_1_/TiN, only linearly adsorbed CO molecules at 2080 cm^−1^ could be detected, while for Pt catalysts with nanoclusters and nanoparticles, bridge-bonded CO molecules at 1820 cm^−1^ were observed [113]. The bridge-bonded configuration of CO molecules needs two adjacent Pt atoms and can only be detected on Pt nanoparticles. Therefore, the bridge-bonded CO peak could be used to exclude the presence of nanoclusters and nanoparticles. With O_2_ as the probe molecule, a low-temperature FTIR experiments showed that single nuclear Fe_1_-N-C sites mainly show a superoxo-like vibration with a O-O bond length of 1.34 Å, while binuclear Fe_2_-N-C are dominated with peroxo-like adsorption with a O-O bond length of 1.48 Å, suggesting that Fe_2_-N-C provides more favorable adsorption configuration to activate oxygen molecules and shows high intrinsic ORR activity [125].

### 3.7. Nuclear Magnetic Resonance

In nuclear magnetic resonance (NMR) measurements, when nuclei in a strong constant magnetic field are perturbed by a weak oscillating magnetic field, a responding electromagnetic signal with a frequency characteristic of the magnetic field at the nucleus will form. If the oscillation frequency matches the intrinsic frequency of the nuclei, this process occurs near resonance. It should be noted that the oscillation frequency relies on the strength of the chemical environment, the static magnetic field, and the magnetic properties of the involved isotope. In a ^27^Al solid-state magic-angle spinning NMR spectroscopy, Kwak and coworkers found that the pentacoordinate Al^3+^ sites (Al^3+^_penta_) formed on the (100) facets of *γ*-Al_2_O_3_ by dehydration and dehydroxylation at elevated temperatures and the coordinatively unsaturated Al^3+^_penta_ sites function as the anchoring position to bind Pt atoms [126]. With the Pt loadings lower than 1 wt%, each Al^3+^_penta_ interacts with exact one Pt atom and forms atomically dispersed Pt/*γ*-Al_2_O_3_ catalysts.

### 3.8. X-Ray Photoelectron Spectroscopy

X-ray photoelectron spectroscopy (XPS) is a commonly used surface analysis technology to detect the element oxidation state. For SAECs, the central metal element generally coordinates with adjacent heteroatoms (such as N, O, P, S) to decrease the surface energy and form stabilized single-atom configuration. During the course, the central metal would transfer charges into coordinated heteroatoms and show some oxidative states due to the relatively low electronegativity of the central metal. This oxidative state of the central metal in SAECs could be detected by XPS analysis [114]. For example, Han et al. observed that after the formation of single-atom Fe-N_*x*_-C catalyst, the central Fe atom shows higher binding energy than metallic iron and lower binding energy than Fe_2_O_3_ [56], which indicates that the charge transfer from central Fe to adjacent N occurs. Generally, XPS analysis is accompanied with XANES measurements to further confirm the oxidative state of the central metal element.

By using the synchronous illumination X-ray photoelectron spectroscopy (SI-XPS) and the synchronous illumination diffuse reflectance Fourier-transform infrared spectroscopy (SI-DRIFTS), Zhang et al. detected the charge transfer as well as chemical bond evolution of single-atom Pt/C_3_N_4_ under light irradiation [127]. Upon photo-induced charge excitation and transfer, the dynamic variations of Pt-N bond cleavage into Pt^0^ and C-N bond transformation into C=N bond of C_3_N_4_ can be experimentally observed. Such dynamic changes designate Pt^0^ and C_3_N_4_ as the sites for reduction and oxidation reactions separately. However, these transformations could not be detected on the Pt nanoparticles decorated on C_3_N_4_ catalyst. This work highlights the importance of synchronous illumination spectroscopic characterizations to understand the dynamic evolution of catalysts.

### 3.9. Inductively Coupled Plasma Atomic Emission Spectroscopy

Inductively coupled plasma atomic emission spectroscopy (ICP-AES) is a trace analytical technique used to measure the content of chemical elements. The plasma acts as a high-temperature source to excite atoms and ions to emit electromagnetic radiation, which is unique to a specific element. The intensity of the emissions from different wavelengths is proportional to the element contents in the sample. ICP-AES is one of the most commonly used technologies to measure the accurate mass loading of the central metal atoms in SAECs [51, 56]. It must be mentioned that before ICP-AES measurements, suitable methods should be adapted to digest the SAEC samples to guarantee the complete dissolution of solid samples.

### 3.10. Elemental Analysis

Elemental analysis is a commonly used analytical technique to measure certain element contents in organic compounds, such as H, C, and N. Firstly, a sample is burned completely in excess oxygen. Then, the combusted products, such as carbon dioxide, water, and nitric oxide, were collected by various traps. By calculating the masses of these combustion products, the composition of the unknown sample could be obtained. Modern advanced elemental analyzers are competent to measure the content of sulfur along with CHN simultaneously. The elemental analysis technology is usually used to measure the content of N and S in SAECs [128, 129].

### 3.11. Density Functional Theory Calculations

Compared with nanoparticles and nanoclusters, single-atom catalysts possess simple, uniform, and well-defined structures. Therefore, density functional theory (DFT) calculations show great potential in confirming the optimized configuration of single-atom catalysts, elucidating the interaction between active sites and substrate species, and illustrating the specific reaction mechanism. For example, single-atom Fe-N-C catalyst showed high ORR activity with a half-wave potential up to 0.900 V [51]. The 4e^−^ ORR reaction mechanism in the alkaline medium is as follows, where ∗ represents the active site:

- O_2_(g) + ∗⟶O_2_∗
- O_2_∗+H_2_O(l) + e^−^⟶∗OOH + OH^−^
- ∗OOH + e^−^⟶∗O + OH^−^
- ∗O + H_2_O(l) + e^−^⟶∗OH + OH^−^
- ∗OH + e^−^⟶OH^−^ + ∗

DFT calculations show that step (v), i.e., charging of the adsorbed ∗OH to form OH^−^, is the rate determining step and compared with Fe nanoparticles the higher activity of Fe-N-C stems from the facile electron transfers from Fe single atom to the adsorbed ∗OH species. The DFT calculation results matched well with that of the electronic chemical potential calculations. Single-atom Zn-N-C catalyst exhibited competitive ORR activity and better ORR durability than that of Fe-N-C in both acidic and alkaline electrolytes. DFT calculation demonstrated that different from Fe-N-C, the pyridinic-N in Zn-N-C was less prone to be protonated in the acidic solutions [130]. Meanwhile, DFT calculation also reveals that the Zn-N_4_ moiety is more electrochemically stable than the Fe-N_4_ in the ORR process. Therefore, Zn-N-C with a high Zn loading of 9.33 wt% shows excellent ORR performance. Atomically dispersed Zn-Co bimetallic catalyst (Zn/Co-N-C) shows a theoretical overpotential of 335 mV during ORR process. DFT calculations reveal that compared with Zn-N-C and Co-N-C, bimetallic Zn/Co-N-C shows superior oxygen binding ability, which greatly lengthens the O-O bond from 0.123 to 0.143 nm and promotes the cleavage of O-O bond [131].

Han and coworkers systematically calculated the adjacent effect between two neighboring Fe-N-C sites of isolated single-atom FeN_*x*_ (*x* = 3 or 4) sites on ORR performance [132]. It was found that the adsorption energies of O_2_ and OH are closely relevant to the density, the reactant coverage, and the active-site coordination. Due to the electron transfer from two Fe atoms to O_2_, the adsorption of O_2_ or OH is enhanced by the adjacent FeN_3_ site. Shortening the Fe-Fe distance results in stronger binding and activation of O_2_. The increased FeN_3_ density decreases the activation energy barrier of the ORR potential determining step (PDS). In contrast, the adjacent effect is weaker on FeN_4_, except for the extremely dense FeN_4_-FeN_4_ (4.1 Å) model. The reason for the difference between FeN_3_ and FeN_4_ is that FeN_3_ sites show stronger magnetic exchange interaction and stronger intersite communication than those of FeN_4_ sites. Further investigation on the impact of active-site density on the kinetics of ORR with Fe-N-C single-atom catalysts is highly expected.

## 4. SAECs for Oxygen Reduction Reactions

**Figure 7:**
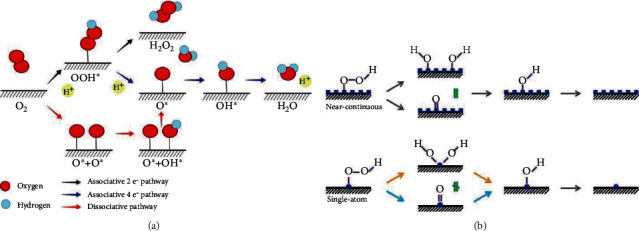
Oxygen reduction reaction mechanism. (a) Oxygen reduction reaction pathway associated with 2e^−^ or 4e^−^ process. Reproduced from [134]. (b) O∗ and 2OH∗ ORR mechanisms on near-continuous and SAECs (blue route: on Co-based SAECs; orange route: on Fe-based SAECs). Reproduced from [135].

ORR holds a special place in the field of electrocatalysis and is the heart of many electrochemical energy storage and conversion systems. As shown in Figure 7(a), ORR involving multiple reaction steps proceeds by either a two-electron (2e^−^) or a four-electron (4e^−^) pathway, depending on different catalysts and electrolytes [16, 133, 134]. The 2e^−^ ORR pathway mainly used for Li-O_2_ battery and production of H_2_O_2_, while the 4e^−^ ORR pathway is favorable for metal-air batteries and low-temperature fuel cells due to its high energy conversion efficiency. As shown in Figure 7(a), it is commonly accepted that the associative 4e^−^ ORR follows an O∗ mechanism (O_2_⟶∗OOH⟶∗O⟶∗OH⟶H_2_O) and the adsorption-free energies of ∗OOH, ∗O, and ∗OH follow the scaling relations. This O∗ mechanism could correctly predict the half-wave potential of Co-based SAECs but underestimated that of Fe-based SAECs. Zhong and Li found that on Fe-based SAECs, the associative 4e^−^ ORR follows an unusual 2OH∗ mechanism (O_2_⟶∗OOH⟶2OH∗⟶∗OH⟶H_2_O) [135]. As shown in Figure 7(b), for traditional nanoparticle- and nanocluster-based catalysts featured with near-continuous active sites, the free energy of O∗ equals that of 2OH∗ due to the free energy scaling relations (Δ*G*(O∗) = 2Δ*G*(OH∗) = Δ*G*(2OH∗)), while for Fe-based SAECs, the scaling relations follow an unconventional equation: Δ*G*(2OH∗) = Δ*G*(O∗) + 1.5 eV. Therefore, 2OH∗ ORR mechanism was proposed and could predict the half-wave potential of Fe-based SAECs well.

### 4.1. Oxygen Reduction Reaction via 2e^−^ Pathway

**Figure 8:**
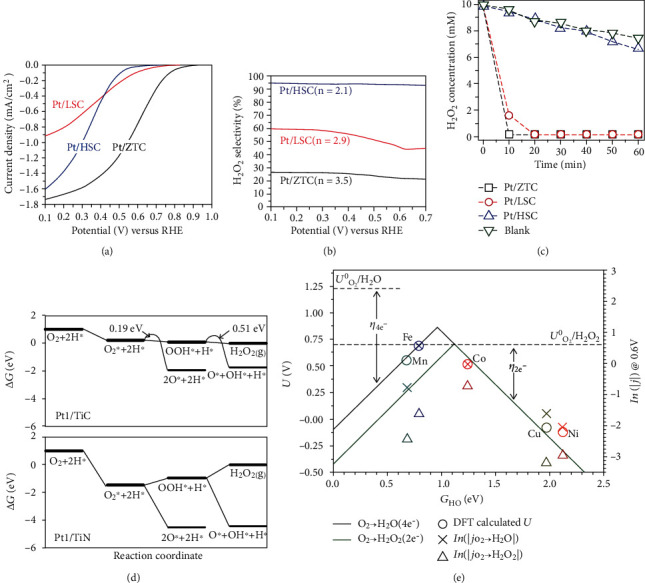
Oxygen reduction reaction via 2e^−^ pathway on SAECs. (a) ORR polarization curves of prepared catalysts. Reproduced from [34]. (b) Selectivity towards H_2_O_2_ measured with RRDE. Reproduced from [34]. (c) Changes of H_2_O_2_ concentration. Reproduced from [34]. (d) Comparison of 2e^−^ ORR-free energy diagrams on Pt/TiN(100) and Pt/TiC(100). Reproduced from [113]. (e) Volcano curves for 2e^−^ (green solid line) and 4e^−^ (black solid line) ORR. Reproduced from [137].

The selectivity towards 2e^−^ or 4e^−^ pathway is critically influenced by the capability of the electrocatalyst to break the O-O bond in the ORR process. The lower capability to cleaving the O-O bond is, the higher selectivity towards 2e^−^ ORR is. In principle, cleaving the O-O bond requires two adjacent active sites to simultaneously adsorb an oxygen molecule, weaken the strength of the O-O bond, and finally break the O-O bond. In this context, SAECs with atomically isolated sites show great potential for inhibiting the cleavage of the O-O bond and achieving high selectivity towards 2e^−^ ORR. When atomically isolated Pt atoms were anchored on *N*-vacancy of TiN (Pt_1_/TiN), in comparison with Pt nanoparticle catalysts, the selectivity of Pt single atoms toward H_2_O_2_ reached to 53.1% [136]. For Pt nanoparticle catalysts, two adjacent Pt-Pt atoms facilitate the adsorption of Pt-O-O-Pt, which causes the O-O bond easily be cleaved, and ORR follows the 4e^−^ pathway. With the Pt nanoparticles further dispersed into isolated Pt atoms, the ORR on Pt_1_/TiN was altered to 2e^−^ pathway; in that, the metal-support interactions changed the adsorption energy toward O_2_ and thus weakened the capability for cleaving the O-O bond. The electron transfer path would be varied depending on their support and coordination environment. When isolated Pt atoms were stabilized on *C*-vacancy of TiC (Pt_1_/TiC), the 2e^−^ ORR selectivity further increased to 68% due to the weaker affinity of oxygen species on Pt_1_/TiC that preserved the O-O bond compared with that of TiN [113]. As shown in Figure 8, promising H_2_O_2_ selectivity of up to 92%-96% over a wide potential range was achieved by coordinating single Pt atoms with S atoms due to the strong Pt-S interaction [34]. Coordinating with S induced the distortion of the Pt center off the square planar geometry due to the more favorable interaction with water molecules [48].

DFT modeling predicted that compared with pure Au, isolated single atoms of Pd, Pt, or Rh stabilized in the Au lattice would elevate the 2e^−^ ORR selectivity. Jirkovský et al. prepared a Au_1-__*x*_Pd_*x*_/C catalyst by using a sequential reduction method [138]. The Au_1-__*x*_Pd_*x*_/C catalyst with a Pd concentration of 8% showed the best 2e^−^ ORR performance with the 2e^−^ ORR selectivity of 95%. The presence of isolated Pd atoms within Au nanoparticle surface enhanced in 2e^−^ ORR selectivity, whereas surface ensembles of contiguous Pd atoms are benefit for 4e^−^ ORR. Further DFT calculations predicted that Pt-Hg with isolated active sites may function as an active, selective, and durable catalyst for 2e^−^ ORR [18].

Transition-metal single-atom electrocatalysts (M-N-C) are mainly investigated to catalyze 4e^−^ ORR to H_2_O, while their applications in 2e^−^ ORR to H_2_O_2_ remain limited. Sun and coworkers investigated a series of M-N-C (M=Fe, Co, Ni, Mn, and Cu) by combining experiments and theoretical calculations to illustrate the rules in electrocatalytic H_2_O_2_ production [137]. Among them, single-atom Co-N-C exhibited the best performance on H_2_O_2_ production in terms of high ORR activity, highest H_2_O_2_ selectivity, and lowest H_2_O_2_ reduction reaction activity. DFT calculations demonstrated that Co-N-C catalyst showed a binding energy of HO∗ intermediates approaching the top of the volcano curve suggesting favorable 2e^−^ ORR. Wang and coworkers revealed that a carbon-supported single-atom Ni catalyst with a tetradentate NiN_2_O_2_ coordination structure could also provide excellent 2e^−^ ORR performance with a H_2_O_2_ selectivity of 96% [117].

### 4.2. Oxygen Reduction Reaction via 4e^−^ Pathway

Besides the 2e^−^ ORR pathway, most reported SAECs were used for the 4e^−^ ORR due to the high energy conversion efficiency in advanced batteries systems. The electrocatalytic activity of SAECs for the 4e^−^ ORR is closely related with the central metal, the ligand, the active-site density, and the pore structures of the support.

#### 4.2.1. SAECs with Single-Metal Sites

**Figure 9:**
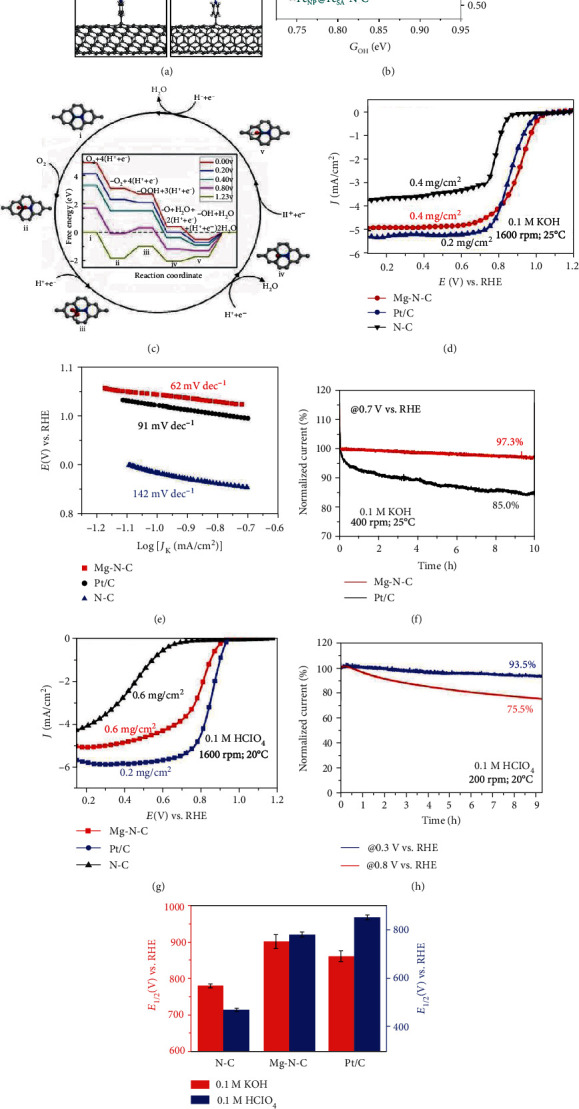
Oxygen reduction reaction via 4e^−^ pathway on SAECs with single metals. (a) Comparison of adsorbed O-containing species. Reproduced from [38]. (b) ORR overpotentials calculated on different samples. Reproduced from [139]. (c) ORR mechanism on the single-atom g-P-N_1_-Pt_1_ catalyst. Reproduced from [140]. (d) ORR polarization curves of as-prepared catalysts measured in alkaline solution. Reproduced from [67]. (e) Tafel slopes of as-prepared catalysts. Reproduced from [67]. (f) Durability tests of Mg-N-C in alkaline solution. Reproduced from [67]. (g) ORR polarization curves of as-prepared catalysts measured in acid electrolyte. Reproduced from [67]. (h) Durability tests of Mg-N-C in acid electrolyte. Reproduced from [67]. (i) Comparison of half-wave potentials for as-prepared catalysts. Reproduced from [67].

So far, several kinds of transition metal-based (Fe, Co, Cu, Zn, Mn, Cr, Mo, Pt, Ru, etc.) and main-group metal-based (Mg, Al, Ca, etc.) SAECs have been developed. Among the reported SAECs, Fe-based SAECs showed the highest ORR activities in alkaline electrolytes in terms of half-wave potential and limit current density. XAS and DFT calculations confirm that atomically dispersed Fe atom-coordinated four N centers (FeN_4_) are the commonly reported active sites for 4e^−^ ORR [51]. Axially coordinated Cl atoms to the FeN_4_ center (Cl-Fe-N_4_) would inhibit the adsorption of oxygen molecules and resulted in decreased ORR activity [141]. On the contrary, axially coordinated N and O atoms to the FeN_4_ center would enhance ORR activity [142]. Cao et al. used a covalent tethered method to immobilized Fe phthalocyanine (FePc) onto 4-aminopyridine-functionalized carbon nanotubes (CNTs) to prepare single-atom Fe electrocatalyst (FePc-Py-CNTs) by axial coordination [38]. The FePc-Py-CNT catalyst shows a half-wave potential of 0.915 V (vs. RHE), superior to that of FePc-CNTs and the benchmark commercial 20 wt% Pt/C catalyst. As shown in Figure 9(a), compared with four ligated FePc-CNTs, the axial coordination of the Py group to FePc in five ligated FePc-Py-CNTs causes the rehybridization of Fe 3d orbitals. As a result, the electronic and geometrical configuration of the FePc-Py-CNT with five ligands totally differs from those of the FePc-CNT with four ligands. On five-ligated FePc-Py-CNT, O-O bonds show higher stretching degree, which induces the cleavage of oxygen molecules easier. In addition, the five-ligated FePc-Py-CNT shows higher charge density near Fermi level, which facilitates the ORR process. The extra axial coordination bond between Fe and the Py group in FePc-Py-CNT suppresses the Fe ion dissociation and thus improves the durability.

The intrinsic activity of single-atom Fe-N-C sites could be further boosted by simultaneously formed metallic Fe nanoclusters or Fe_3_O_4_ nanoparticles. With a 1,3,5-tris(4-aminophenyl)benzene and terephthaldehyde-based covalent organic frameworks (TAPB-PDA COF) as the host, Ao et al. fabricated a novel ORR electrocatalyst by integrating Fe atomic clusters in a Fe-N-C matrix (Fe_AC_@Fe_SA_-N-C) [139]. The half-wave potential of Fe_AC_@Fe_SA_-N-C reaches 0.912 V (vs. RHE) in 0.1 M KOH, superior to that of pure single-atom Fe_SA_-N-C counterpart (0.844 V), most reported platinum-group free electrocatalysts, and even commercial Pt/C (0.897 V). Both experiments and theoretical calculations elucidate that the ORR activity of Fe_AC_@Fe_SA_-N-C originates from active Fe-N-C sites but is remarkably boosted by the Fe nanoclusters, as shown in Figure 9(b). Wang and coworkers further pointed out that Cu nanoparticles encapsulated in the carbon shell could improve the ORR kinetics of Fe-N-C with the ORR Tafel slope decreasing from 86 to 78 mV dec^−1^ [143]. Besides FeN_4_ sites, FeN_2_, CoN_2_, CoN_4_, ZnN_4_, and CuN_3_-vacancy sites also exhibit high efficiency for 4e^−^ ORR in alkaline electrolytes [52, 87, 144, 145].

Different from alkaline solutions, the 4e^−^ ORR in acidic electrolytes proceeds through either a dissociative route or an associative route, depending on the energy barrier of the oxygen dissociation (O_2_ + 2∗⟶2O∗) step on the catalyst surface. For SAECs, the atomically dispersed M-N-C sites restrain the cleavage of the O-O bond and show high oxygen dissociation barrier. As a result, the 4e^−^ ORR on SAECs in acidic electrolytes follows an associative mechanism [146], where ∗ represents the active site:

- O_2_ (g) + ∗⟶O_2_∗
- O_2_∗+H^+^ + e^−^⟶∗OOH
- ∗OOH + H^+^ + e^−^⟶∗O + H_2_O(l)
- ∗O + H^+^ + e^−^⟶∗OH
- ∗OH + H^+^ + e^−^⟶H_2_O(l) + ∗

At the present stage, the ORR performance of SAECs in acid electrolytes is less satisfactory, owing to instability and slower kinetics. Different from other Pt-based single-atom catalysts, a Pt_1_-N/BP shows a 4e^−^ ORR process with a half-wave potential of 0.76 V in 0.1 M HClO_4_ and a much lower H_2_O_2_ yield [140]. As shown in Figure 9(c), pyridinic-N-immobilized Pt atoms serve as the active sites, totally different from those on commercial Pt/C catalysts. Consistent with DFT calculations that the single-atom Ir-N-C catalyst would exhibit the nearest Δ*G*_OH∗_ to the apex of the classic volcano plot, the as-prepared Ir-based SAEC catalyst shows a half-wave potential of 0.864 V outperforming most of reported alternatives to Pt in acidic electrolyte and displays a record high turnover frequency (TOF) of 24.3 e^−^ site^−1^ s^−1^ and mass activity of 12.2 A mg^−1^_Ir_ at 0.85 V in 0.1 M HClO_4_ [147]. Zhang et al. prepared a single-atom Ru-based electrocatalyst (Ru-N/G-750), which showed onset and half-wave potential of 0.89 and 0.75 V, respectively, in 0.1 M HClO_4_, along with better stability and poisoning tolerance to MeOH and CO than commercial Pt/C. Quantitative analysis of XAFS indicated the formation of Ru-N_4_ moieties embedded in graphene sheets with axial oxygen adsorption (Ru-oxo-N_4_). Theoretical calculations revealed that ORR activity of Ru-N/G-750 originated from the Ru-oxo-N_4_ moieties rather than Ru-N_4_ moieties [148]. Xiao et al. further reported that a Ru-based SAEC (Ru-SSC) showed unprecedented TOF up to 4.99 e^−^ s^−1^ site^−1^, far surpassing the benchmark Fe-SSC counterpart (0.816 e^−^ s^−1^ site^−1^) [149].The excellent ORR activity of Ru-SSC could be attributed to the suitable OH∗ adsorption-free energy. In addition, Ru-SSC showed low Fenton reactivity, which enables the Ru-SSC much better durability (only 17 mV negative shift after 20000 cycles) than the corresponding Fe-SSC catalyst (31 mV).

Besides noble metal-based SAECs, Fe-based SAECs also showed high ORR activity in acidic electrolyte [150]. However, Fe-based SAECs show undesirable properties for application in PEMFCs; in that, Fe^2+^ or Fe^3+^ ions derived from Fe-based SAECs could react with H_2_O_2_ and produce hydroxyl and hydroperoxyl radical species. The attack of free radicals to the PEMFC membrane severely degrades the performance and even leads to the cell failure [151]. As a result, Pt- and Fe-free high-performance catalysts are highly desirable to improve the ORR performance in acidic media. For example, the unexpected Fenton reaction could be substantially reduced on single-atom CrN_4_ sites [152]. The ORR activity of the single-atom M-N-C catalysts follows the order of Fe > Co > Mn > Cu > Ni in acid electrolytes. Therefore, Co seems to be the most promising alternative transition metal to substitute Fe [153]. By optimizing the synthetic conditions, a Co-based single-atom catalyst (20Co-NC-1100) showed a half-wave potential of 0.8 V in 0.5 M H_2_SO_4_ [63], which is comparable to Fe-based catalysts and only 60 mV lower than commercial Pt/C. By utilizing the confinement of surfactants to increase the CoN_2+2_ active-site density, the single-atom Co-N-C@F127 catalyst increased the half-wave potential to 0.84 V in 0.5 M H_2_SO_4_ [62]. It has been found that Mn atoms could catalyze the graphitization of the organic precursor in the carbonization process and thus Mn dopant in the carbon matrix enhances the stability of resultant nanocarbon skeleton, which promotes the stability of Mn-based SAECs. Li et al. developed a two-step doping and adsorption approach to prepare single-atom Mn-N-C catalyst [154]. This method is effective to significantly increase the active-site (MnN_4_ moieties) density. The as-prepared Mn-N-C exhibited a high half-wave potential up to 0.80 V and remarkable durability in acid electrolytes, due to the ultrastable MnN_4_ sites and the improved corrosion resistance of adjacent carbon by Mn doping. First principle calculations showed that the MnN_4_C_12_ site possesses a favorable binding energy with oxygen containing species, such as O_2_, OOH, and H_2_O, as well as a surmountable energy barrier to break O-O bonds for the 4e^−^ ORR.

In generally, transition metal-based SAECs are regarded as the efficient ORR catalysts due to the suitable center position of d band, while main-group metals, such as Mg, Al, and Ca, are considered to be catalytic inactive, particularly for ORR with multistep O-containing species conversion. In contrast to transition-metal center with a narrow d-band, the main-group metal sites show a delocalized s/p-band, which endows the adsorbate with broadened states. The interaction between the adsorbates and the unsuitable surface leads to too weak (no activation) or too strong (poison to the active sites) adsorption. However, Mg cofactors in enzymes show extremely high activity for biochemical reactions and Mg-centered chlorophyll plays vital roles in photosynthesis. Liu and coworkers for the first time used the main-group metal (Mg) to construct the Mg-N-C single-atom electrocatalyst for alkaline 4e^−^ ORR [67]. The Mg-N-C catalyst was prepared by pyrolyzing Mg-based metal organic framework (Mg-HMT) followed by acid pickling. As shown in Figure 9(d), the single-atom Mg-N-C catalyst shows high ORR activity with half-wave potential (*E*_1/2_) of 0.91 V and onset potential (*E*_onset_) of 1.03 V in 0.1 M KOH, which surpasses the performance of commercial Pt/C and far exceeds that of most transition metal-based catalysts reported so far. In addition, the single-atom Mg-N-C catalysts exhibit comparable ORR activity to Pt/C with half-wave potential (*E*_1/2_) of 0.79 V. DFT calculations indicated that a higher p-state location formed when a Mg center coordinated with two N atoms (MgN_2_ configuration) in the graphene matrix in comparison with MgN_3_ and MgN_4_, which weakens the binding strength of O-containing species at Mg centers and results in an activity near the top of volcano curves.

#### 4.2.2. SAECs with Binary Metal Sites

Inspired by biological heme-copper oxidases playing critical roles in the 4e^−^ ORR in biosystems [155], binary metal-based SAECs (denoted as M_1_-M_1_-N-C or M_1_-M_2_-N-C) attracted more and more attention for ORR. DFT calculations indicate that the formation of the atomically isolated binary metal sites would facilitate the dual-site adsorption and cleavage of the O-O bond, reduce the adsorption barrier for the activation of oxygen molecules, and boost the ORR activity [156].

A binary metal SAEC (Fe,Mn-N/C) was prepared to mimic the heme-copper oxidases (HCOs) by adsorbing FeSO_4_ and manganese(II) 2,4-pentanedionate (C_10_H_14_MnO_4_) into the pores of ZIF-8 followed by pyrolyzing at 900°C [59]. Fe,Mn-N/C embeds two types of M-N_*x*_ sites (Fe-N_*x*_ and Mn-N_*x*_) in the porous graphene framework and shows remarkably high ORR activity with a half-wave potential of 0.904 V and a kinetic current density of 33.33 mA cm^−2^, which is 4.9 times higher than that of commercial Pt/C (6.76 mA cm^−2^). Compared with single-metal catalysts (Fe-N/C and Mn-N/C) and nonmetal catalyst (N/C), the activity sequence for the ORR process is as follows: Fe,Mn-N/C > Fe-N/C >> Mn-N/C ≈ N/C, which indicates that Fe-N_*x*_ sites function as the active center in the ORR process, Mn-N_*x*_ sites serve as the assistant center, and the synergistic effect between Fe-N_*x*_ sites and Mn-N_*x*_ sites remarkably boosts the ORR activity of the binary metal Fe,Mn-N/C. First principle calculations revealed that the synergistic effect between Fe-N_*x*_ sites and Mn-N_*x*_ sites dramatically lowers the protonating energy barrier from O∗ to OH∗ in the ORR process compared with single-metal Fe-N/C.

**Figure 10:**
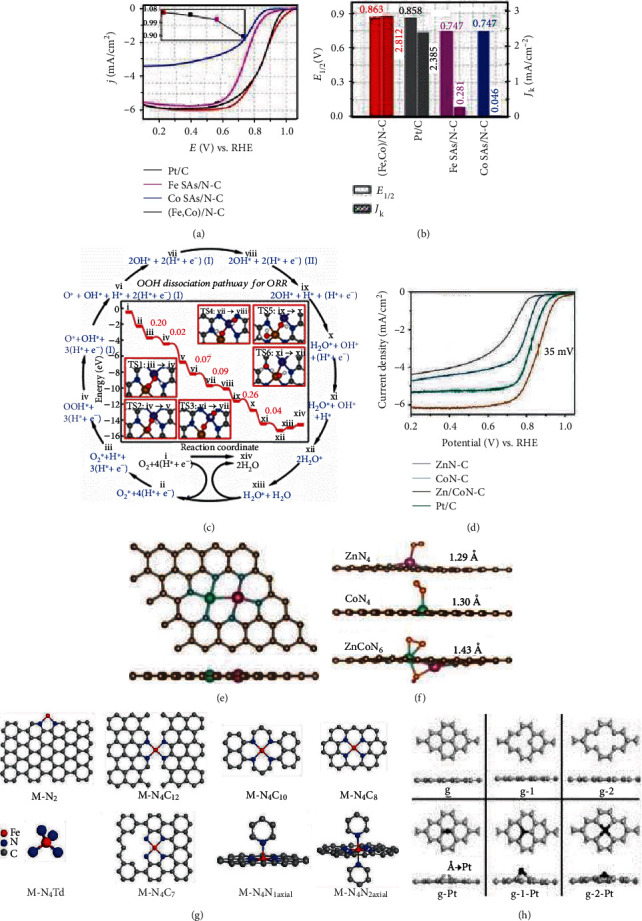
Oxygen reduction reaction via 4e^−^ pathway on SAECs with binary metals and single ligands. (a) ORR LSV curves of as-prepared catalysts in acid electrolyte. Reproduced from [123]. (b) Comparison of half-wave potential and *J_K_*. Reproduced from [123]. (c) ORR mechanism on (Fe,Co)/N-C. Reproduced from [123]. (d) ORR polarization curves of as-prepared catalysts in alkaline solution. Reproduced from [131]. (e) Optimized structure of Zn/CoN-C. Reproduced from [131]. (f) Comparison of adsorbed O_2_ on different active centers. Reproduced from [131]. (g) List of reported Fe-N_*x*_-C_*y*_ configurations. Reproduced from [160]. (h) Calculated models of different substrates. Reproduced from [161].

In acidic electrolyte (0.1 M HClO_4_), as shown in Figure 10(a), a binary metal (Fe,Co)/N-C catalyst showed a high ORR activity with a half-wave potential of 0.867 V and an onset potential (*E*_onset_) of 1.06 V, higher than those of commercial Pt/C [123]. As the cathode catalyst, (Fe,Co)/N-C possessed outstanding ORR durability. After 50000 CV cycles, no obvious decay could be detected. Theoretical calculations elucidated that the dissociation barriers of O_2_ and OOH into O and OH on binary Fe-Co SAs/N-C sites were as low as 0.25 and 0.02 eV, respectively, much lower than those on single Fe SAs/N-C or Co SAs/N-C sites, as shown in Figure 10(c). The strong binding of oxygen molecules on Fe-Co binary sites promoted the cleavage of O-O bond.

The initial ORR performance of single-atom Fe-N-C catalysts exceeds other ORR catalysts and even comparable with Pt/C catalysts, whereas under harsh reaction conditions, especially in acid electrolytes, the durability of Fe-N-C catalysts is unsatisfactory [157]. Four kinds of primary deactivation mechanisms for the Fe-N-C catalysts have been put forward: (1) leaching of Fe species, (2) protonation of coordinated nitrogen atoms, (3) being attacked by OH radicals derived from H_2_O_2_ decomposition by Fenton reaction, and (4) flooding over the active sites. Zeng and coworkers reported a precise modulation of Fe-N-C catalyst at the atomic level by a “single-atom to single-atom” anchoring of a Pt atom onto the Fe atom at FeN_4_ centers through a bridging oxygen molecules and formed the Pt_1_@Fe-N-C catalyst with a rebuilt active moiety Pt_1_-O_2_-Fe_1_-N_4_ [158]. The Pt_1_@Fe-N-C with a Pt loading of 2.1 wt% showed a half-wave potential of 0.8 V in 0.5 M H_2_SO_4_. It was found that the introduction of Pt_1_-O_2_- onto the Fe-N-C did not increase the ORR activity but significantly improved durability of Fe-N-C. The improved durability of Pt_1_@Fe-N-C was attributed to the alleviated H_2_O_2_ attack; in that, Pt_1_-O_2_- cap could inhibit or disturb the Fe ions to catalyze the Fenton reaction; the contents of OH radicals would be greatly lowered and the oxidation of the active sites would be mitigated. In addition, the decreased OH concentration also relieved the electrooxidation of the carbon surface and micropore flooding. The Pt_1_-O_2_- cap could slightly reduce the Fe centers of Fe-N-C, which made the reduced Fe center a longer ionic radius and made the Fe-N coordination more stable.

Besides heteronuclear bimetal SAECs, SAECs with homonuclear metal pairs have also been reported to display outstanding ORR performance. By pyrolysis of a bimetallic ZnCo-ZIF with a Zn/Co ratio of 10 at 900°C, Xiao et al. for the first time revealed the homonuclear binary Co_2_N_5_ configuration in the carbon matrix [159]. The binuclear Co_2_N_5_ site shows a half-wave potential of 0.79 V in 0.1 M HClO_4_ solution. The mass activity of the Co_2_N_5_ site is up to 7468 mA mg_Co-Co_^−1^, nearly 12 times higher than the single nuclear CoN_4_ site. After 20000 continuous CV cycles in accelerated durability test, the *E*_1/2_ only negatively shifted 12 mV on the Co_2_N_5_ electrode, suggesting the remarkably high stability of the binuclear Co_2_N_5_ site in acidic electrolyte. DFT calculations elucidate that the novel binuclear Co_2_N_5_ site shows remarkably lowered thermodynamic barrier towards the adsorption and activation of ORR intermediates and thus exhibits greatly boosted intrinsic activity. Ye et al. prepared a series of Fe_1_-N-C, Fe_2_-N-C, and Fe_3_-N-C electrocatalysts by in situ encapsulating Fe(acac)_2_, Fe_2_(CO)_9_, and Fe_3_(CO)_12_ in the nanocavity of ZIF-8 during the synthesis of ZIF-8 followed by pyrolysis at 800°C [125]. The binary nuclear Fe_2_-N-C catalyst shows excellent ORR activity with a half-wave potential of 0.78 V (vs. RHE) in 0.5 M H_2_SO_4_ solution as well as remarkable durability with only a negative 20 mV shift after 20000 CV cycles, which are much better than the conventional single nuclear Fe_1_-N-C catalyst. DFT calculations and low-temperature FTIR of O_2_ adsorption experiments confirmed that Fe_1_-N-C mainly shows a superoxo-like vibration with a O-O bond length of 1.34 Å, while Fe_2_-N-C are dominated with peroxo-like adsorption with a O-O bond length of 1.48 Å, suggesting that Fe_2_-N-C provides more favorable adsorption configuration for the activation of oxygen molecules. High-resolution XPS demonstrates that compared with the Fe_1_-N-C sites, the Fe_2_-N-C sites are more conducive to the formation of pyridinic N in the carbon skeleton. The rich pyridinic N species would further enhance the ORR activity.

#### 4.2.3. SAECs with Single Ligands

Excellent electron donating properties of N atoms make N-doped carbon frameworks suitable candidates to stabilize atomically isolated metal atoms and form SAECs. N ligands doped in the carbon matrix could coordinate with various isolated metal atoms, including Fe, Co, Zn, Cu, Mn, Cr, Ru, Pt, Ir, and Mg, with different coordination numbers and different configurations, which results in the obtained SAECs showed different ORR activities.

Due to unique empty d orbital structure, atomically dispersed Fe atoms could feature various types of coordination structures and numerous M-N_*x*_-C_*y*_ SAECs have been reported [11, 160, 162], including Fe-N_2_, Fe-N_4_-C_8_, Fe-N_4_-C_10_, Fe-N_4_-C_12_, Fe-N_4_ Td, Fe-N_5_, and Fe-N_6_. In most cases, single-atom Fe tended to form planar Fe-N_4_ moieties by coordinating with four pyrrolic or pyridinic N atoms (Fe-N_4_-C_12_ or Fe-N_4_-C_10_, respectively) after treating the Fe, N, and C precursors at high temperature (above 800°C). The Fe-N_4_ moiety exhibits a half-wave potential of 0.900 V (vs. RHE) [51], which is 58 mV higher than that of commercial Pt/C (0.842 V). Shen et al. reported that the FeN_2_ site outperformed the FeN_4_ site in ORR process, due to its weaker interaction with ∗O_2_ and ∗OH intermediates and improved electron transport [145]. Compared with the Fe-O bonding length (*d*_Fe-O_) and the O-O bonding length (*d*_O-O_) on O_2_-adsorbed FeN_4_ site, the shorter *d*_Fe-O_ and the longer *d*_O-O_ on the FeN_2_ one indicated that the adsorption and dissociation of O_2_ molecules on FeN_2_ sites is more rapid than on the FeN_4_ sites. Similarly, the Co-N_2_ centers showed higher ORR activity with a half-wave potential of 0.881 V than those of Co-N_4_ (0.863 V) and even commercial Pt/C (0.811 V) [52]. Co-N_2_ centers interacted more strongly with peroxide than Co-N_4_ centers and showed superior 4e^−^ ORR performance. For Mg-based SAECs, the Mg-N_2_ configuration serves as the ORR active sites and shows a half-wave potential of 0.91 V and onset potential of 1.03 V in 0.1 M KOH [67]. Compared with MgN_3_ and MgN_4_, the MgN_2_ configuration weakens the binding strength of O-containing species at Mg atom and results in an activity approaching the top of the volcano curve. For Cu-based SAECs, the Cu-N_3_-vacancy structure functions as the ORR active sites and provides a half-wave potential of 0.92 V with a kinetic current density of 8.87 mA cm^−2^ at 0.9 V in alkaline electrolyte [87].

Besides N ligands, Liu et al. revealed that C atoms could also be used to anchor isolated single Pt atoms (Pt_1.1_/BP_defect_) [161]. EXAFS experiments and DFT calculations elucidated that isolated Pt atoms were stabilized by four carbon atoms in carbon divacancies (PtC_4_). The carbon-defect-anchored Pt-based SAEC (Pt_1.1_/BP_defect_) showed high ORR activity in 0.1 M HClO_4_ electrolyte with the half-wave potential and limit current density higher than those of commercial Pt/C catalysts. DFT calculations revealed that isolated Pt atoms showed strong charge transfer to coordinated carbon atoms and the formed PtC_4_ centers function as the active sites to adsorb and activate oxygen-related intermediates to accelerate the ORR rate.

#### 4.2.4. SAECs with Binary Ligands

**Figure 11:**
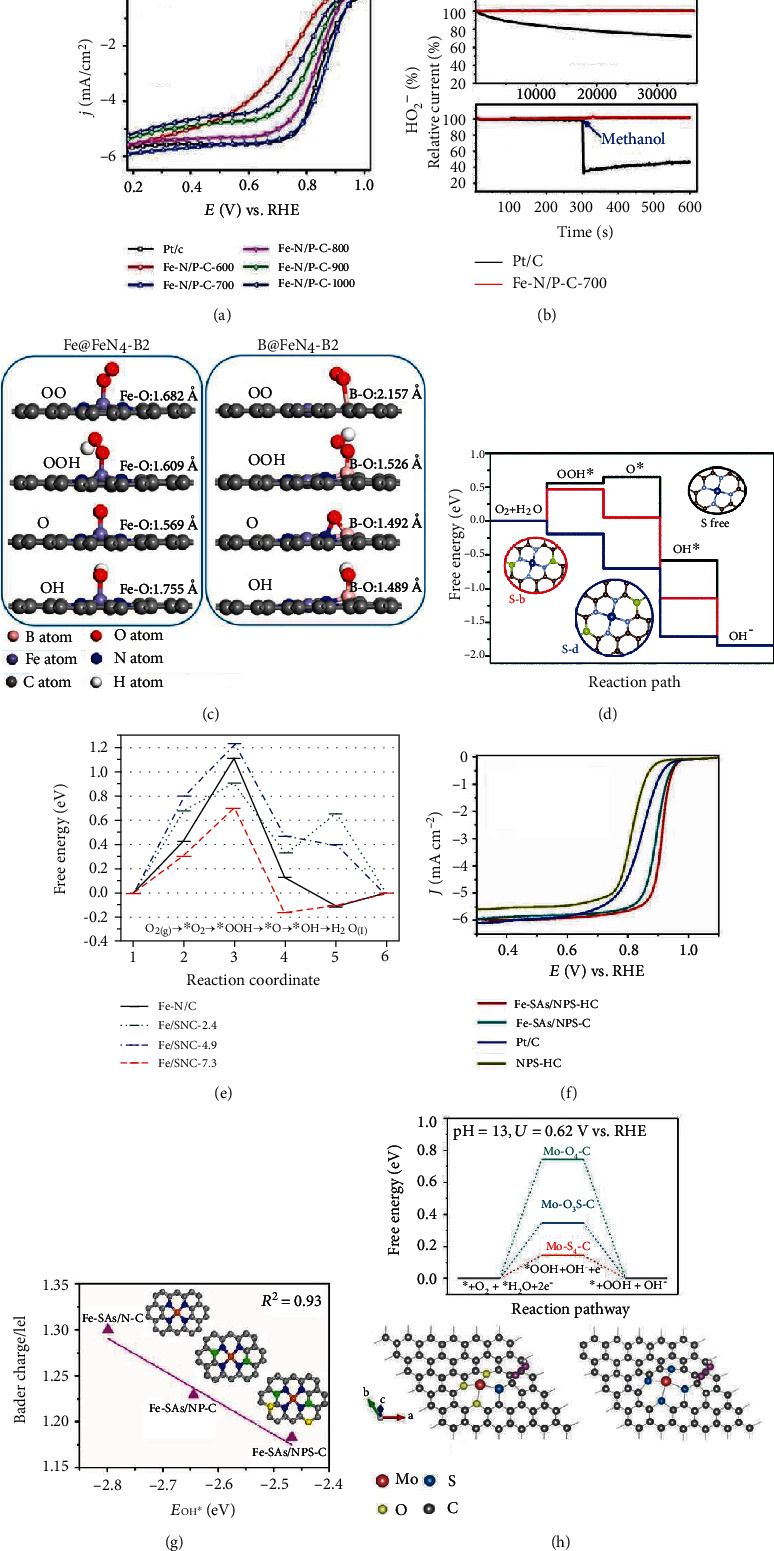
Oxygen reduction reaction via 4e^−^ pathway on SAECs with binary ligands. (a) LSV curves of as-prepared catalysts in alkaline solution. Reproduced from [77]. (b) Durability and methanol tolerance tests of as-prepared catalysts. Reproduced from [77]. (c) Proposed ORR mechanism on FeN_4_-B_2_. Reproduced from [55]. (d) DFT calculated ORR-free energy diagram on different Cu-based catalysts. Reproduced from [164]. (e) ORR-free energy diagram on as-prepared catalysts. Reproduced from [165]. (f) LSV curves of N, S, and P codoped single-atom Fe catalysts. Reproduced from [60]. (g) Linear relationship between Bader charge and OH∗ binding energy of prepared single-atom Fe catalysts. Reproduced from [60]. (h) ORR mechanism on single-atom Mo catalysts. Reproduced from [166].

As early as 1964, Jasinski has revealed that cobalt phthalocyanine (CoPc) could catalyze the ORR [50]. Since then, tremendous efforts have been devoted to designing and synthesizing SAECs featuring various types of metal centers and coordination shells. In general, two strategies are adopted to tune the electronic structure of SAECs: (i) directly modulating the ligands coordinated to the central metals and (ii) using long-range interactions between doped heteroatoms (e.g., B, C, N, O, P, S) on the second or higher coordination shells of the substrate and metal centers. For instance, as shown in Figure 11(a), compared with single N ligand FeN_4_ sites, the binary N and P ligand-coordinated FeN_3_P sites showed higher ORR activity in both alkaline and acidic electrolytes [77]. DFT calculations revealed that the N and P binary-coordinated iron sites provided suitable energy barrier for oxygen intermediate activation and thus accelerated the reaction kinetics. With triphenylphosphine (PPh_3_) as the P-containing precursor, Jin and coworkers for the first time reported that P atoms could directly coordinate with the Fe-N_*x*_ sites and form a C-P-Fe-N_*x*_-P-C configuration, which showed an ultrahigh ORR activity with a half-wave potential up to 0.923 V and an ORR Tafel slope of 56 mV dec^−1^ in alkaline media [163]. In contrast to directly coordinating with central Fe atoms, the introduction of boron centers in the carbon matrix could also improve the ORR activity of the FeN_*x*_ sites [55]. DFT calculation suggests that the incorporation of boron dopant greatly decreased the ORR energy barrier, as shown in Figure 11(c).

Due to the different electronegativities between O and N atoms, coordinated oxygen atoms could endow novel electronic structure and electrochemical property of isolated central metal atoms compared with traditional N atoms. Yang et al. successfully incorporated O and N atom-coordinated Mn cofactors within the conductive 3D porous graphene matrix as biomimetic electrocatalysts for ORR [167]. Mimicking the coordinative effect of electronic interaction between O, N, and Mn atoms in enzymes, the energy level of the Mn d-electrons could be tuned to a reasonable state. The calculated results showed that Mn-N_1_O_3_, Mn-N_2_O_2_, and Mn-N_3_O_1_ are possible ORR active sites. Moreover, the downshifted position of d-band center and adjacent position of first peak relative to Fermi level in Mn-N_3_O_1_ cofactor is beneficial for desorption and formation of ORR intermediates, enabling the fastest ORR kinetics, which demonstrates that coordinated O atoms play a critical role in modulating the intrinsic activity of transition metals in specific structures. In alkaline medium, the as-prepared single-atom Mn/C-NO catalyst exhibits the highest ORR activity among reported Mn-based electrocatalysts and even better than Pt/C catalyst.

Besides B, O, and P dopants, S atom is another kind of commonly used dopant to tune the ORR activity of SAECs. Li et al. designed a pyrrole-thiophene copolymer pyrolysis approach to decorate single Fe atoms on S and N codoped porous carbon catalyst (Fe-ISA/SNC) [72]. The ORR activity of Fe-ISA/SNC shows a volcano-type profile with the increase of sulfur contents and the optimal Fe-ISA/SNC with 1 : 1 N/C ratio showed a half-wave potential of 0.896 V, which surpasses Fe single atoms on nitrogen-doped carbon (Fe-ISA/NC, 0.839 V), commercial Pt/C (0.841 V), and most reported nonnoble metal catalysts. XAFS experiments confirmed that FeN_4_S_2_ moieties serve as the active sites. DFT calculations suggested that relative low electronegativity of sulfur could enrich the charge on nitrogen atoms, which promoted the rate-determining adsorption of OH∗ and accelerated the entire ORR process. Another binary S and N codoped Fe-based single-atom catalyst (Fe/SNC) showed a high half-wave potential of 0.77 V towards the ORR with superior methanol tolerance and Pt/C-comparable stability in acidic media [165]. The incorporated sulfur into the carbon skeleton generates a thiophene-like structure (C-S-C). Although the C-S-C sites are less active for the ORR in acidic media, as shown in Figure 11(e), they play a significant part in decreasing the electron localization around the Fe centers, lower the ORR activation barrier on Fe-N-C in acidic electrolyte, and assist the reduction of intermediate H_2_O_2_. The synergistic effects between C-S-C and Fe-N-C sites boost the ORR activity, as manifested by a positive shift in the half-wave potential of 30 mV when compared with the sulfur-free Fe-N-C counterpart. Chen et al. reported a similar sulfur dopant improved Fe-based SAECs for ORR in alkaline electrolyte [168]. Apart from toxic organic precursors, Jin et al. reported that in the presence of hydrazine hydrate, the sulfate ions could act as the inorganic sulfur source to form C-S-C bonds in the Fe-N-C-decorated carbon matrix [169, 170]. They also found that sulfuration of Fe-N-C embedded with Fe_*x*_C/Fe species could generate the Fe-S bonds at the interface, which could remarkably enhanced the ORR performance in acidic media with a half-wave potential of 0.821 V and without deactivation after 10000 CV cycles [171]. Jiang et al. reported that sulfur dopant in the carbon matrix could also enhance the ORR activity of Cu-based SAECs (Cu-SA/SNC) [164]. As shown in Figure 11(d), XAS analysis together with DFT calculations confirms that the excellent ORR activity stems from the strong synergistic interaction at the atomic interface between Cu atoms and the N and S codoped carbon supports, leading to the reduction of the reaction-free energy for the intermediate adsorption. In situ experiments showed that bond-shrinking low-valence Cu (+1)-N_4_-C_8_S_2_ moieties at the atomic interface of Cu-SA/SNC play significant roles in an oxygen reduction process. Zhang et al. synthesized a series of porous N and S codoped carbon framework-supported SAECs (M-SAs/NSC, M=Fe, Co, Ni) [172]. Although M-SAs/NSC were prepared through the same synthetic method, Fe-SAs/NSC contains a FeN_4_S_2_ configuration (with S bonding to N atoms), which is totally different from those of Co-SAs/NSC embedded with CoN_3_S_1_ and Ni-SAs/NSC integrated with NiN_3_S_1_ (with S bonding to metal atoms). The ORR activity follows the order of Fe-SAs/NSC > Co-SAs/NSC > Ni-SAs/NSC. DFT calculations reveal that the higher charge density and the lower energy barriers at FeN_4_S_2_ sites account for the higher ORR catalytic activity of Fe-SAs/NSC.

Furthermore, Kirkendall effect usually used to construct yolk shell nanostructures was adopted to fabricate single-atom Fe sites embedded in hollow carbon polyhedron doped with N, S, and P by pyrolysis of a MOF@polymer precursor [60]. The electronic properties of single-atom Fe sites were modulated by near-range coordination with N and long-range interaction with S and P. The as-prepared Fe-SAs/NPS-HC catalyst offers a half-wave potential of 0.912 V (vs. RHE), a kinetic current density of 71.9 mV cm^−2^ at 0.85 V, and a Tafel slope of 36 mV dec^−1^ that is record level among previously reported ORR catalysts in alkaline electrolyte, as shown in Figure 11(f). Fe-SAs/NPS-HC also shows outstanding ORR performance in acidic electrolyte with an *E*_1/2_ of 0.791 V, close to that of Pt/C and higher than most reported nonprecious metal catalysts. To be highlighted, Fe-SAs/NPS-HC possesses superior electrochemical durability. DFT calculations reveal that high efficiency and favorable kinetics of Fe-SAs/NPS-HC could be attributed to atomic dispersion of FeN_*x*_ moieties and electronic modulation from the interaction with decorated S and P atoms, which transfers charges to isolated Fe centers to enrich the charge of Fe (Fe^*δ*+^) centers and thus weaken the binding of adsorbed OH species.

The introduction of the second ligands could enhance both the 4e^−^ ORR and the 2e^−^ ORR process. A binary O and S ligand-coordinated Mo-based SAECs (Mo_1_/OSG) could catalyze 2e^−^ ORR with a high H_2_O_2_ selectivity above 95% in 0.1 M KOH [166]. The Mo_1_/OSG exhibited a high onset potential (0.78 V vs. RHE for -0.1 mA cm^−2^), large diffusion-limiting disk current density (-2.78 mA cm^−2^ at 0.30 V), and low Tafel slope of 54.7 mV dec^−1^ close to the theoretical limit for 2e^−^ ORR. The calculated H_2_O_2_ selectivity on Mo_1_/OSG is above 95% and the electron transfer number is approximately 2.1 in a wide range of potentials, suggesting a high selectivity to 2e^−^ ORR, which is higher than reported catalysts such as atomic Fe-O_*x*_-C (95%), edge site-rich nanocarbon (90~95%), oxidized carbon nanotube (90%), B-C-N material (80~90%), and atomic Co-N_*x*_-C (80~90%) in alkaline media. In contrast, the H_2_O_2_ selectivity of binary O and S codoped graphene (OSG) is only 35% with an electron transfer number of 3.3, indicating that isolated single Mo atoms play crucial roles in producing H_2_O_2_. The H_2_O_2_ reduction reaction (H_2_O_2_RR) current density of Mo_1_/OSG is less than -0.1 mA cm^−2^ when the potential is more positive than 0.40 V (vs. RHE), revealing poor activity towards H_2_O_2_ reduction on the Mo-O/SC active sites. As shown in Figure 11(h), DFT calculations suggested that compared with single O ligand-coordinated single Mo sites, the binary O and S-coordinated single Mo sites showed enhanced adsorption to the critical ∗OOH intermediates, indicating a much facilitated reaction thermodynamics. In addition, N and O-coordinated single-atom Ni center (NiN_2_O_2_) supported on carbon could also provide excellent 2e^−^ ORR performance with a H_2_O_2_ selectivity of 96%.

#### 4.2.5. SAECs with Unique Support Structures

**Figure 12:**
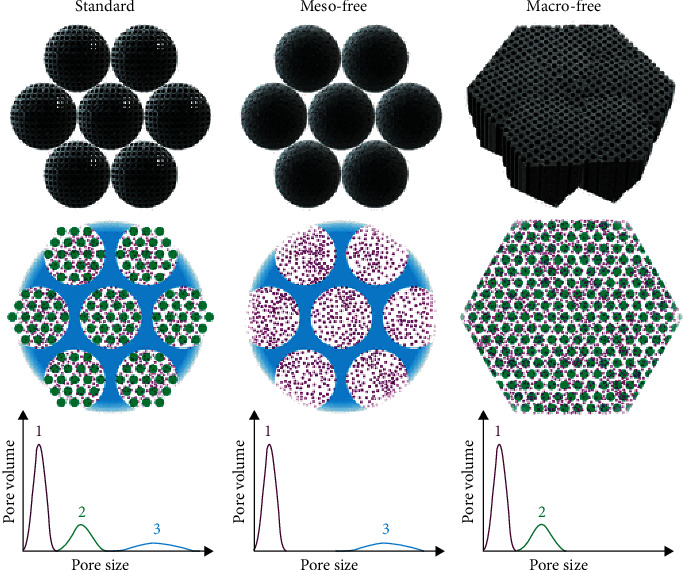
Proposed catalysts with different pore structures. Reproduced from [173].

SAECs have exhibited great potential in electrocatalysis. Previous efforts concentrate on elevate catalytic activity by simply increasing metal loadings, which, however, might induce the aggregation of metal species to form inactive metal nanoparticles. Engineering support morphology and porosity to enhance mass transport and promote the utilization efficiency of active sites is another effective approach to improve ORR performances. As shown in Figure 12, meso- and macropores with low mass transfer resistance facilitate macromolecules' transportation. As a result, substrates could contact the active sites anchored in the entire catalyst particles and thus the ORR efficiency could be promoted [173]. Constructing suitable support materials with unique structures for SAECs favors to disperse isolated metal atoms and accelerates the charge transfer kinetics in the electrocatalytic processes. An ideal SAEC support should possess strong coordination to anchor single-metal atoms, large specific surface area to accommodate active sites, and superior electrical conductivity to transport electrons.

Since the pioneering work reported by Liang and coworkers that silica nanoparticles could be used as the template to prepare single-atom Co-N-C catalysts by adsorption and pyrolysis of Vitamin B_12_ [174], various hard templates such as self-assembled polystyrene sphere (PS) [150, 175], silica colloidal crystal [153, 176–178], silica particles with a hollow core and a mesoporous shell [114], self-assembled Fe_3_O_4_ nanocubes [179], and ordered mesoporous silica [145] have been used to construct SAECs with atomically dispersed active sites embedded in the carbon framework with hierarchical pores from micropores to mesopores and macropores to increase the accessible active-site numbers and enhance the mass and electron transports. Lee and coworkers further systematically investigated the influence of a porous carbon structure on the ORR activity by preparing three types of N-doped carbon catalysts with different pore size distributions but similar Brunauer-Emmett-Teller (BET) surface area and active-site concentration [173]. They found that mesopores and macropores account for different stages of the reaction kinetics: (i) Mesopores contribute to electrolyte wetting of the physical surface area to increase the portion of electrochemically available active sites. (ii) Macroporous structure promotes kinetic accessibility to the available active sites in the time scale of ORR. (iii) The standard catalyst with optimized hierarchical ordered porous structure from micropores to mesopores and macropores showed the highest ORR activity in both alkaline and acidic electrolytes.

**Figure 13:**
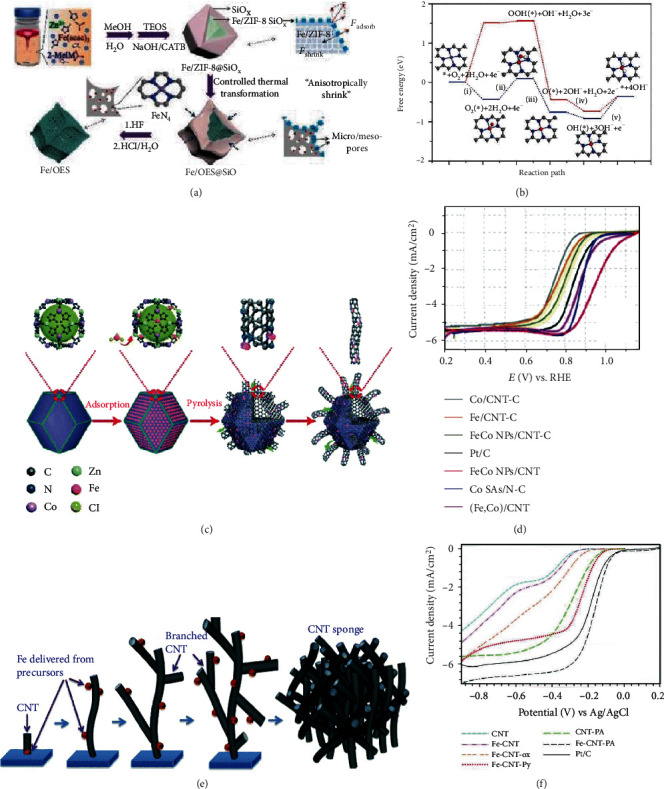
Oxygen reduction reaction via 4e^−^ pathway on SAECs with unique structures. (a) Preparation of single-atom Fe/OES. Reproduced from [61]. (b) Comparison of ORR-free energy diagrams. Reproduced from [61]. (c) Synthesis of the single-atom (Fe,Co)/CNT. Reproduced from [180]. (d) LSV curves of as-prepared catalysts in alkaline solution. Reproduced from [180]. (e) Preparation of CNT sponge. Reproduced from [181]. (f) LSV curves of as-prepared catalysts in alkaline electrolyte. Reproduced from [181].

As shown in Figure 13(a), Hou et al. fabricated an overhang-eave structured support embedded with isolated iron sites with a silica-mediated method [61]. The as-prepared Fe/OES catalyst showed superior ORR performances to traditional bulk carbon structure-supported single-atom Fe catalysts (Fe/BCS). This overperformed ORR activity stems from its edge-rich structure, which offers abundant three-phase boundaries to enhance substrate mass transport and improve the accessibility of single-atom iron sites. With a similar strategy, Wan et al. prepared a Fe-based SAEC (TPI@Z8(SiO_2_)-650-C) with a concave-shaped external surface structure and high Fe-N_*x*_ density [182]. This unique concave structure guarantees high utilization of the densely dispersed Fe-N_4_ moieties and greatly lower mass transport limitations.

As shown in Figures 13(c) and 13(d), a carbon nanotube- (CNT-) supported Fe-Co binary metal single-atom electrocatalyst ((Fe,Co)/CNT) provides unexpectedly high ORR activity with an admirable onset potential of 1.15 V and a half-wave potential of 0.954 V (vs. RHE) [180], which are the highest values reported. Excellent conductivity of CNTs, unique 2D nanosized structure of CNTs for fully accessible active sites, and the synergistic effect between FeN_*x*_ and CoN_*x*_ dramatically boost the ORR activity of (Fe,Co)/CNT.

**Figure 14:**
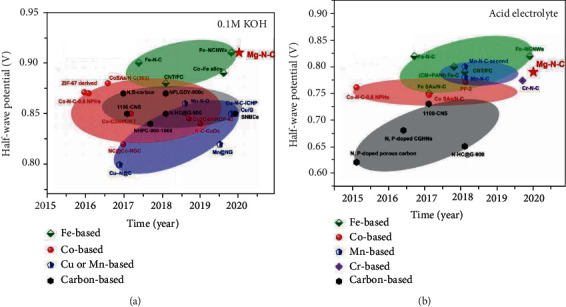
ORR activity comparison. (a) Comparison of typical ORR electrocatalysts in 0.1 M KOH alkaline electrolyte. Reproduced from [67]. (b) Comparison of typical ORR electrocatalysts in acid electrolytes. Reproduced from [67].

As shown in Figure 13(e), Yang et al. prepared a free-standing 3D carbon nanotube (CNT) sponge-supported single-atom Fe catalyst (Fe-CNT-PA) by first growth Fe-containing 3D CNT sponges and then introduction of N dopants [181]. Fe-CNT-PA exhibited strikingly improved ORR activity and stability in acidic and alkaline electrolytes, with the onset potential and limiting current density comparable or superior to those of commercial Pt/C catalyst, as shown in Figure 13(f). No obvious deactivation was detected after accelerated stress tests (AST) up to 30000 CV cycles, demonstrating its excellent long-term durability of Fe-CNT-PA. The outstanding ORR performance of Fe-CNT-PA could be attributed to the porous sponge-like self-standing 3D CNT structure, which substantially enhances the mass transfer and elevates the utilization efficiency of accessible active sites.  Figure 14 collected and compared ORR performance in both alkaline and acidic electrolytes of typical electrocatalysts reported in very recent years.

## 5. ORR-Based Applications of SAECs

High performance of single-atom electrocatalysts on ORR process encouraged researchers to use SAECs for various systems in energy conversion and storage as well as fine chemical synthesis. Generally, oxygen reduction reactions involve 2e^−^ ORR and 4e^−^ ORR. The involved applications include Li-O_2_ battery with nonaqueous organic electrolytes, zinc-air batteries, and anion exchange membrane fuel cells with aqueous alkaline electrolytes, hydrogen peroxide production and proton exchange membrane fuel cells with aqueous acidic electrolytes, and microbial fuel cells with aqueous neutral electrolytes.

### 5.1. Production of Hydrogen Peroxide (H_2_O_2_)

Hydrogen peroxide (H_2_O_2_) is a widely used fine chemical for water treatment, pulp bleaching, medical care, and chemical synthesis. The annual demand for hydrogen peroxide has reached up to 4 million tons. Currently, the production of hydrogen peroxide is primarily through the costly and energy-intensive anthraquinone process. An attractive and appealing alternative route for direct on-site production of H_2_O_2_ is through an electrochemical process in a fuel cell setup (anode: H_2_ = 2e^−^ + 2H^+^; cathode: O_2_ + 2e^−^ + 2H^+^ = H_2_O_2_, *E*^0^ = 0.695 V), where ORR occurs via a 2e^−^ pathway. In general, oxygen reduction reactions involve two routes: 2e^−^ ORR and 4e^−^ ORR. The selectivity towards 2e^−^ or 4e^−^ pathway is determined by the capability of the electrocatalyst to cleave the O-O bond during the ORR process. In principle, to increase the selectivity of H_2_O_2_ production through ORR, the O-O bond cleavage needs to be minimized. Breaking of the O-O bond required two adjacent active sites to simultaneous adsorb an oxygen molecules. SAECs featuring with atomically isolated active sites facilitate the end-on adsorption mode for oxygen molecules, other than peroxo mode, which lowers the possible O-O bond cleavage. In this context, SAECs would be appropriate for H_2_O_2_ production via 2e^−^ ORR.

**Figure 15:**
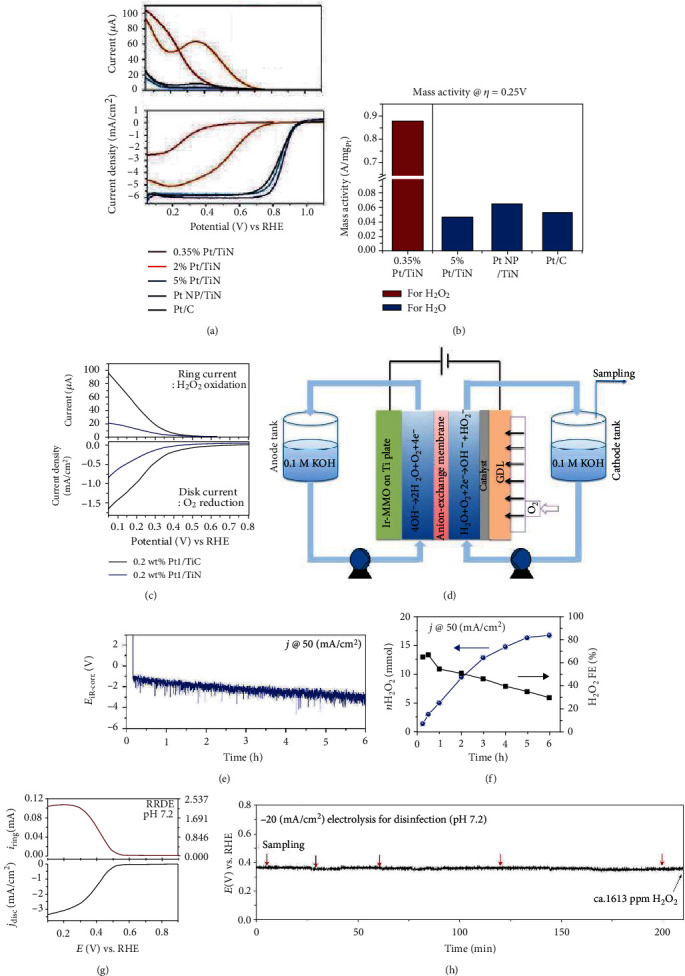
Production of hydrogen peroxide with SAECs. (a) LSV curves and ring currents in acid electrolyte. Reproduced from [136]. (b) Comparison of mass activity for different Pt catalysts. Reproduced from [136]. (c) Comparison of ring currents on different Pt catalysts. Reproduced from [113]. (d) Schematic illustration of the microflow cell. Reproduced from [137]. (e) Durability test of the microflow cell setup. Reproduced from [137]. (f) Productivity and Faradic efficiency for H_2_O_2_. Reproduced from [137]. (g) ORR polarization curves of Fe-CNT in neutral electrolyte. Reproduced from [183]. (h) Durability test of Fe-CNT catalyst. Reproduced from [183].

Compared with Pt nanoparticles, a single-atom Pt_1_/TiN catalyst with a Pt loading of 0.35 wt% showed higher selectivity towards H_2_O_2_ with a mass activity of 78 A g^−1^ at an overpotential of 50 mV [136], as depicted in Figures 15(a) and 15(b). Isolated single-atom Pt active sites play a crucial part in achieving high H_2_O_2_ selectivity via ORR. Two neighboring active sites can activate an oxygen molecule and finally cleave the O-O bond. Traditional Pt-based nanoparticle catalysts adopt a 4e^−^ ORR route, while single-atom Pt catalysts follow a 2e^−^ ORR route to produce hydrogen peroxide without break the O-O bond. By replacing TiN with TiC, the single-atom Pt_1_/TiC exhibited improved 2e^−^ ORR performance for electrochemical H_2_O_2_ production [113]. Theoretical calculations illustrated that oxygen species show strong affinity to Pt_1_/TiN and probably poison catalyst surface, while Pt_1_/TiC preserves O-O bonds with high selectivity toward H_2_O_2_ production. Single Pt atoms on sulfur-doped zeolite-templated carbon (Pt/HSC) dominantly provide a 2e^−^ pathway for the H_2_O_2_ production with a selectivity of 96% [34]. The S-moieties embedded in the carbon framework coordinate with single Pt atom to form a four-coordinated mononuclear Pt complex (PtS_4_), which functions as the active site for H_2_O_2_ production with high selectivity in acid conditions. A high-concentration (24.8 at%) single-atom Pt catalyst (h-Pt_1_-CuS_*x*_) with embedded Pt atoms in amorphous hollow CuS_*x*_ spheres could directly reduce oxygen into H_2_O_2_ with a selectivity of 92~96% over a wide potential range in acidic electrolyte [48]. An H_2_O_2_ kinetic current of 35 A g_cat_^−1^ was achieved at 0.4 V, which is nearly one order of magnitude higher than that of those previous reported single-atomic Pt catalysts. The strong coordination between Pt and S enables the h-Pt_1_-CuS_*x*_ showing excellent stability. In an electrochemical device that directly produces H_2_O_2_ from hydrogen and oxygen molecules, the H_2_O_2_ productivity reached up to 546 mol kg_cat_^−1^_h_^−1^.

Ye and coworkers investigated the relationship between the structure of transition metal (Mn, Fe, Co, Ni, and Cu) single-atom electrocatalysts anchored in nitrogen-doped graphene and the catalytic performance of hydrogen peroxide (H_2_O_2_) synthesis via electrochemical 2e^−^ oxygen reduction reaction (ORR) [125]. The cobalt single-atom catalyst (Co SAC) showed optimal ∗OOH adsorption energy and provided a high H_2_O_2_ production rate, even higher than the noble metal electrocatalysts. The kinetic current for H_2_O_2_ production reached 1 mA cm^−2^_disk_ at 0.6 V (vs. RHE) with H_2_O_2_ faraday efficiency above 90%. Durability tests showed that no decay was observed after 10 h. Both experiments and theoretical calculations show that the Co SAC show optimized d-band center and can act as a highly efficient electrocatalyst for H_2_O_2_ production. After systematically investigating a series of M-N-C materials (M=Mn, Fe, Co, Ni, and Cu), Sun and coworkers also revealed that single-atom Co-N-C catalyst showed the best H_2_O_2_ productivity considering its high ORR activity, highest H_2_O_2_ selectivity, and lowest H_2_O_2_RR activity [137]. DFT calculations demonstrated that the HO∗ binding energy over Co-N-C catalyst is close to the top of the volcano curve indicating favorable 2e^−^ ORR. As shown in Figure 15(d), in a microflow cell device, the industrial H_2_O_2_ productivity reached an unprecedented production rate of more than 4 mol peroxide g_catalyst_^−1^ h^−1^ at a current density of 50 mA cm^−2^. A carbon-supported single-atom Ni catalyst (Ni-N_2_O_2_/C) with a tetradentate NiN_2_O_2_ coordination structure was prepared by pyrolysis of Ni-coordinated Jacobsen's ligand at 300°C in Ar [117]. The Ni-N_2_O_2_/C catalyst provided outstanding 2e^−^ ORR performance for the electrocatalytic reduction of oxygen molecules to H_2_O_2_ in 0.1 M KOH with a H_2_O_2_ selectivity of 96%. A H_2_O_2_ production rate of 5.9 mol g_catalyst_^−1^ h^−1^ was achieved at a current density of 70 mA cm^−2^ and the catalyst could be stably maintained over 8 h. To be highlighted, air could be used as the oxygen sources and be electrocatalytically reduced into H_2_O_2_ with a selectivity above 90% at a high current density.

Single-atom Fe-N-C is a well-known 4e^−^ ORR electrocatalyst. By finely modulating oxygen reduction routes on various transition metal (Pd, Fe, Co, and Mn) coordination environments in carbon nanotube, Jiang et al. found that single-atom Fe-O-C is an efficient electrocatalyst for H_2_O_2_ production. Fe-O-C catalyst could offer an onset potential of 0.822 V (vs. RHE) in 0.1 M KOH at 0.1 mA cm^−2^ H_2_O_2_ current and provide a H_2_O_2_selectivity > 95% [183], as shown in Figures 15(g) and 15(h). Assisted by a gas diffusion layer (GDL) electrode, the H_2_O_2_ production rate over Fe-O-C reached up to 43 mA cm^−2^ with a 95.4% selectivity under 0.76 V.

### 5.2. Lithium-Oxygen Batteries (LOBs)

The rechargeable Li-O_2_ battery represents an amazing energy storage and conversion device considering its high theoretical energy storage capacity (11140 Wh kg^−1^). The discharge reactions in Li-O_2_ batteries include (i) Li ⟶ Li^+^ + e^−^ and (ii) O_2_ + 2Li^+^ + 2e^−^ ⟶ Li_2_O_2_, while the charge reactions in Li-O_2_ batteries involve (i) Li_2_O_2_ ⟶ O_2_ + 2Li^+^ + 2e^−^ and (ii) Li^+^ + e^−^ ⟶ Li. The introduction of active cathode catalyst could reduce both discharging and charging overpotentials and improve the overall energy storage efficiency. From a rational design viewpoint, an ideal cathode catalyst in Li-O_2_ batteries should have highly active catalytic centers and dense active-site distribution to achieve maximum catalytic performance. Abraham and Jiang for the first time reported a polymer electrolyte-based rechargeable Li-O_2_ battery with single-atom CoPc (cobalt phthalocyanine) as the catalyst [184]. Compared with the bare carbon support, CoPc catalyst elevated the discharge voltage by 350 mV and lowered the charge overpotential by 300 mV. Consequently, the overpotential between the charge and the discharge is reduced by 650 mV. A soluble heme biomolecule, with an isolated single-atom Fe centers binding with four N atoms, was used as a bifunctional catalyst in Li-O_2_ battery [185]. Heme serves as an oxygen shuttle by accepting and releasing dissociated oxygen species and facilitates battery recharge. The Li-O_2_ battery with heme catalysts achieves a lower polarization and longer cycle life.

**Figure 16:**
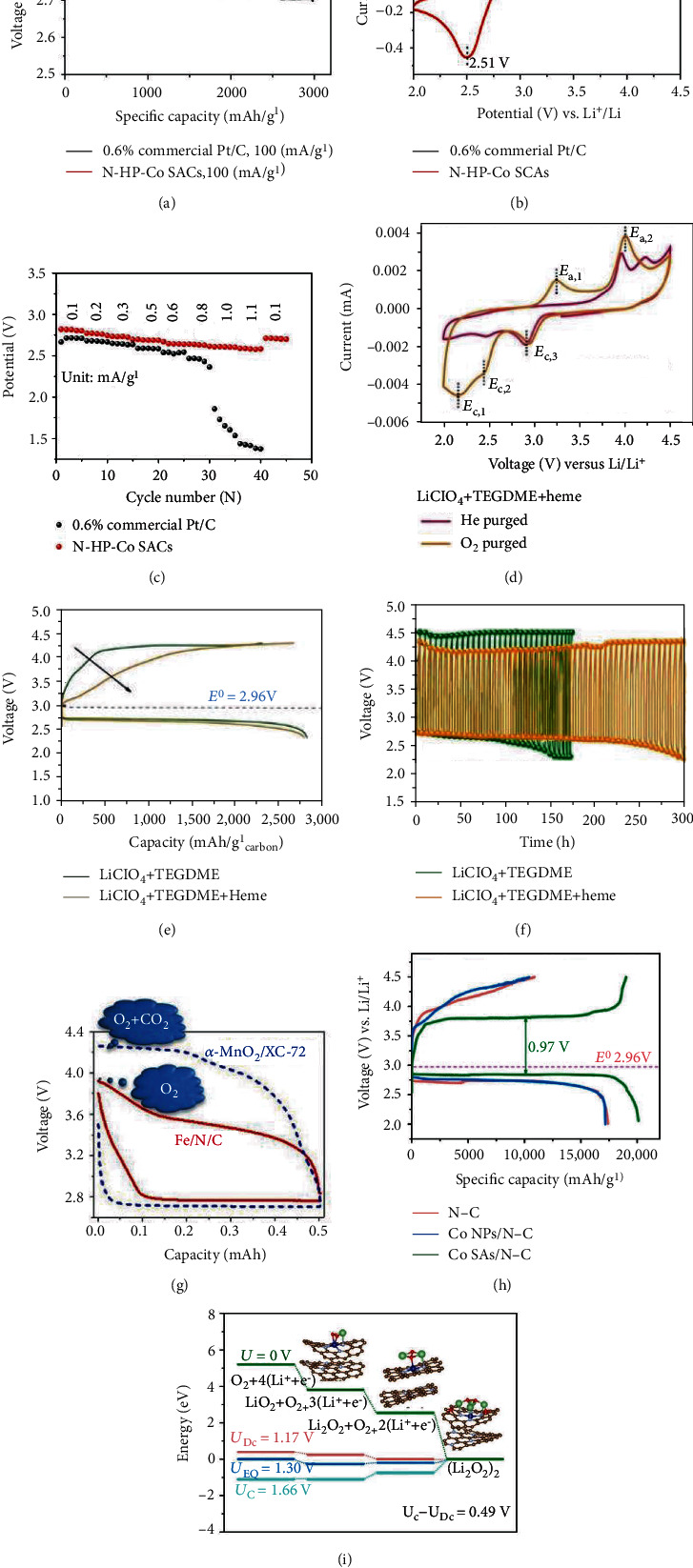
Lithium-oxygen batteries with SAECs as the air cathode catalysts. (a) Comparison of initial discharge voltage of as-prepared catalysts. Reproduced from [186]. (b) CV curves of as-prepared catalysts. Reproduced from [186]. (c) Rate performance tests of Li-O_2_ batteries. Reproduced from [186]. (d) CV curves under different atmospheres. Reproduced from [185]. (e) Comparison of initial cycle performance of the MWCNT electrode in different electrolytes. Reproduced from [185]. (f) Cycling tests of the MWCNT electrodes in different electrolytes. Reproduced from [185]. (g) Charge/discharge curves of Li-O_2_ battery with different catalysts. Reproduced from [187]. (h) The initial cycle performance of Li-O_2_ battery with different catalysts. Reproduced from [89]. (i) DFT calculations for the discharge/charge process on single-atom Co catalyst. Reproduced from [89].

Compared with the well-studied *α*-MnO_2_/XC-72 and bare carbon support, single-atom Fe-N-C exhibited lower charge-discharge overpotential and significant improved Li-O_2_ lifespan [187]. Effluent gas analysis after controlled charge cycles found no carbon dioxide formation with Fe-N-C as the cathode catalyst, indicating that Fe-N-C promoted the decomposition of Li_2_O_2_ over that of the TEGDME electrolyte. Therefore, Fe-N-C-based Li-O_2_ battery showed an enhanced lifespan. Similarly, a bimetallic single-atom FeCu/C catalyst prepared by thermal treatment of FePc and CuPc showed at least 200 mV higher discharge voltage at 0.2 mA cm^−2^ than bare carbon support [188, 189]. In addition, the FeCu/C catalyst could catalyzed the disproportionation of Li_2_O_2_ as ^“^2Li_2_O_2_⟶2Li_2_O + O_2_^”^, which resulted in the decreased fraction of Li_2_O_2_ in the final discharge products. Coupling with the merits of 2D MOFs and atomically dispersed metal sites, the Co-SAs/N-C catalyst could be used as the cathode catalyst for Li-O_2_ battery [89], delivers low-impedance charge transfer pathways, and provides large specific surface area to accommodate Li_2_O_2_. Compared with bare carbon nanosheet (N-C) and cobalt nanoparticle (Co-NPs/N-C) counterparts, uniformly distributed nanosized Li_2_O_2_ species formed on the Co-SAs/N-C electrode during the ORR process. Meanwhile, such nanosized Li_2_O_2_ species play significant parts in the subsequent OER process on CoN_4_ sites, greatly promoting the decomposition of Li_2_O_2_ and suppressing side reactions. As expected, the Co-SAs/N-C electrode provides high discharge voltage (2.85 V vs. Li/Li^+^), remarkably decreased charge/discharge polarization (0.40 V vs. Li/Li^+^), superior discharge capacity (20105 mAh g^−1^ at 200 mA g^−1^ and 11098 mAh g^−1^ at 1 A g^−1^), approximately 100% coulombic efficiency, and remarkably cycling performance (260 cycles at 400 mA g^−1^). As shown in Figure 16(i), abundant Co-N_4_ species acting as active sites can remarkably enhance the intrinsic capability to adsorb lithium superoxide (LiO_2_) and thus control the size, morphology, and distribution of generated Li_2_O_2_. The electrode design facilitates to form nanosized Li_2_O_2_ species during the discharging process and accelerates the decomposition of Li_2_O_2_ species during the charging process due to the intimate contact between Li_2_O_2_ and the active Co-N_4_ sites. Accordingly, redox kinetics and ORR/OER overpotentials are efficiently ameliorated.

### 5.3. Zinc-Air Batteries (ZABs)

With high theoretical energy density (1300 Wh kg^−1^), high safety, and low cost, aqueous zinc-air batteries (ZABs) show great potential for practical applications. In the discharging process, Zn metal is oxidized into Zn(OH)_4_^2-^ at the anode; meanwhile, oxygen molecules are reduced into H_2_O via the 4e^−^ ORR pathway at the cathode. At present, the governing obstacle for the commercial application of ZABs lies in the sluggish ORR kinetics in the cathode discharging process. Normally, Pt-based catalysts are regarded as the state-of-the-art ORR catalysts to accelerate the reaction, whereas high cost, scarce reserves, and unsatisfied durability of Pt significantly impede their large-scale applications. High efficiency, low cost, and remarkable durability of SAECs for ORR in alkaline electrolyte make them promising candidates to serve as the air cathode catalysts for zinc-air batteries.

**Figure 17:**
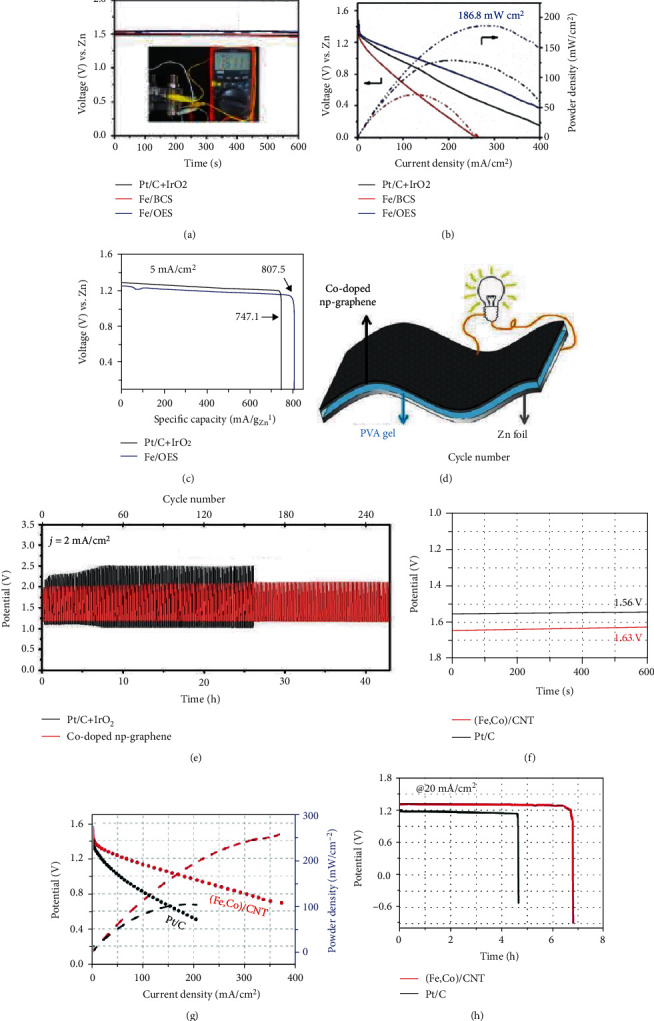
Zinc-air batteries with SAECs as the air cathode catalysts in aqueous alkaline electrolytes. (a) Comparison of open-circuit voltage with different catalysts. Reproduced from [61]. (b) Performance test of Zn-air batteries. Reproduced from [61]. (c) Comparison of the specific capacities. Reproduced from [61]. (d) Schematic illustration of the all-solid-state Zn-air battery. Reproduced from [96]. (e) Cycling test of the all-solid-state Zn-air batteries. Reproduced from [96]. (f) Comparison of open-circuit voltage with different catalysts. Reproduced from [180]. (g) Comparison of Zn-air battery performance with different catalysts. Reproduced from [180]. (h) Galvanostatic discharge profiles of Zn-air batteries. Reproduced from [180].

An overhang-eave-structured carbon support embedded with isolated single-atom iron sites (Fe/OES) was fabricated [61], which shows edge-rich structure with abundant three-phase boundaries to enhance mass transport and increase accessible single-atom iron sites. The Fe/OES catalyst provided an onset potential of 1.0 V (vs. RHE) and half-wave potential of 0.85 V (vs. RHE) in 0.1 M KOH. As shown in Figure 17(a), as the air cathode catalysts, Fe/OES-based zinc-air battery shows an open-circuit voltage (OCV) of 1.5 V. The zinc-air battery with Fe/OES as the cathode catalyst delivers an ultrahigh capacity of 807.5 mAh g_Zn_^−1^ at 5 mA cm^−2^, corresponding to an energy density of 962.7 Wh kg_Zn_^−1^ with Zn utilization of 98.4%. The peak power density of Fe/OES is about 186.8 mW cm^−2^, higher than that of the battery with Pt/C (128.8 mW cm^−2^). Fe/OES-based zinc-air battery stably runs for more than 400 cycles. He et al. also reported that the FeN_*x*_ sites in the Stone-Wales configurations exhibited much lower ORR-free energies than the normal counterparts, which makes the FeN_*x*_ sites an excellent bifunctional (ORR/OER) oxygen electrode with a low voltage gap of only 0.71 V for rechargeable zinc-air batteries [190]. Wu et al. reported a KCl template-assisted method to prepare single-atom Co-based catalyst (SCoNC) with dramatically high Co site fraction of 15.3 % [54]. The SCoNC catalyst with large electrochemically active surface area exhibited high ORR mass activity up to 12.1 A mg_Co_^−1^ at 0.8 V (vs. RHE), nearly an order of magnitude higher than that of the benchmark Pt/C catalyst. The SCoNC-based ZABs offered a peak power density of 194 mW cm^−2^ at 294 mA cm^−2^, higher than those of Pt/C-based cell (177 mW cm^−2^ at 255 mA cm^−2^) under the same conditions. The specific capacity of this device is 690 mAh g_Zn_^−1^ (normalized by the mass of consumed Zn) at a current density of 5 mA cm^−2^ (corresponding to an energy density of 945 Wh kg_Zn_^−1^). A Ni-based single-atom catalyst (codoped np-graphene) by decorating an interconnected nanoporous graphene (np-graphene) with N and Ni atoms was used as the free-standing and flexible air cathode of an all-solid-state zinc-air battery with PVA gel as the electrolyte and Zn foil as the anode [96]. The battery showed an open-circuit voltage of 1.35 V with a low np-graphene loading amount of 0.4 mg cm^−2^. The discharge polarization curves of the codoped np-graphene-based zinc-air battery provide a maximum power density of 83.8 mW cm^−2^, higher than that of the Pt/C-based all-solid-state ZABs (74.5 mW cm^−2^). At 2 mA cm^−2^, the codoped np-graphene-based zinc-air battery stably runs for more than 258 cycles.

Compared with Fe-, Co-, and Ni-based SAECs, Cu-based SAECs as the air cathode catalysts could provide higher peak power density over 200 mW cm^−2^ [191]. Wu et al. prepared a single-atom Cu-based catalyst (Cu-N@C-60) with coordination unsaturated Cu(I)-N active sites embedded in the graphene matrix by pyrolysis of CuPc with dicyandiamide [192]. The graphene matrix provides anchoring sites to stabilize Cu(I)-N sites and serves as the electron-conducting path. To be emphasized, the Cu(II)-N site in CuPc precursor is inert for ORR, while the Cu(I)-N site in Cu-N©C-60 exhibits ultrahigh ORR activity, even comparable to that of 40 wt% Pt/C catalyst. As the air cathode catalysts with a loading of 0.4 mg cm^−2^, Cu-N@C-60-based zinc-air battery provided a current density up to 142 mA cm^−2^ at 1.0 V and the peak power density reaches 210 mW cm^−2^. Zinc-air battery with Cu-N@C-60 shows a high stability. The initial and final battery voltage after 100 h durability test is 1.19 V and 1.16 V at 20 mA cm^−2^, respectively.

As the first reported main-group metal-based SAEC, the Mg-N-C catalyst showed outstanding ORR performance in alkaline electrolyte in terms of high half-wave potential, 4e^−^ ORR selectivity, stability, and methanol tolerance. As the ZAB air cathode, the Mg-N-C catalyst provided a peak power density of 150 mW cm^−2^, a discharge voltage of 1.26 V at 20 mA cm^−2^, and excellent durability [67].

Compared with single-metal SAECs, the synergistic effect between FeN_*x*_ and CoN_*x*_ dramatically boosts the ORR activity of (Fe,Co)/CNT and enable (Fe,Co)/CNT showing unexpectedly high ORR activity with an admirable onset potential of 1.15 V and a half-wave potential of 0.954 V (vs. RHE). As the air cathode, (Fe,Co)/CNT-based zinc-air battery provides an open-circuit voltage as high as 1.63 V, higher than that of Pt/C-based zinc-air battery (1.56 V) [180], as shown in Figure 17(f). The (Fe,Co)/CNT-based ZAB provides a current density of 178 mA cm^−2^ at 1.0 V and a peak power density of 260 mW cm^−2^, greatly exceeding Pt/C-based ZAB. Galvanostatic discharge tests at 20 mA cm^−2^ showed that the voltage of the (Fe,Co)/CNT-based ZAB maintained at 1.31 V, higher than Pt/C-based ZAB. The specific capacity normalized to the mass of consumed Zn for the (Fe,Co)/CNT-based ZAB reached 774 mAh g_Zn_^−1^ at a discharge current density of 50 mA cm^−2^, corresponding to a gravimetric energy density of 870 Wh kg_Zn_^−1^.

The introduction of the second ligand could also significantly improve the performance of SAECs in ZABs. Yuan et al. prepared a binary nitrogen and phosphorus-coordinated single-atom Fe-based catalyst (Fe-N/P-C-700) with FeN_3_P configuration. Compared with single N ligand FeN_4_ sites, the binary N and P ligand-coordinated FeN_3_P sites showed higher diffusion limiting current density of 5.66 mA cm^−2^ and more positive onset potential of 0.941 V and half-wave potential of 0.867 V (vs. RHE). Functioning as the air cathode, Fe-N/P-C-700-based ZAB provides an open-circuit voltage of 1.42 V and a peak power density of 133.2 mW cm^−2^ [77], much higher than that of the Pt/C-based ZAB (74.3 mW cm^−2^). The galvanostatic discharge curve of a Fe-N/P-C-700-based ZAB maintained a steady discharge voltage of 0.93 V and excellent rate capacity ranging from 1 to 100 mA cm^−2^.

### 5.4. Low-Temperature Fuel Cells

A fuel cell is an electrochemical apparatus that converts the chemical energy of a fuel (such as hydrogen, methanol, ethanol, formic acid) and an oxidant (such as oxygen) into electricity through a pair of separated redox reactions. Considering the operating temperatures, fuel cells could be divided into high-temperature fuel cells (such as solid oxide fuel cells operated at temperatures higher than 800°C) and low-temperature fuel cells (such as anion exchange membrane fuel cells, proton exchange membrane fuel cells, microbial fuel cells). Atomically isolated metal atoms in SAECs tend to aggregate at high temperatures above 800°C. Hence, SAECs with excellent ORR performance are usually used as the cathode catalysts of low-temperature fuel cells.

#### 5.4.1. Alkaline Anion Exchange Membrane Fuel Cells (AEMFCs)

Anion exchange membrane fuel cells (AEMFCs) use an anion polymer exchange membrane to transport hydroxide anions and separate the cathode and the anode compartments. AEMFCs are commonly used to power space shuttles. In an AEMFC device, the fuel (such as hydrogen or methanol) is furnished and oxidized at the anode, while the oxidant (such as oxygen) is supplied and reduced at cathode separately. The hydroxide ions (OH^−^) produced at cathode penetrate through the polymer exchange membrane, react with the fuel at the anode, and generate current in the external circuit.

Electrochemical reactions with hydrogen fuel are as follows:

- At anode: H_2_ + 2OH^−^ = 2H_2_O + 2e^−^
- At cathode: O_2_ + 2H_2_O + 4e^−^ = 4OH^−^

Electrochemical reactions with methanol fuel are as follows:

- At anode: CH_3_OH + 6OH^−^ = CO_2_ + 5H_2_O + 6e^−^
- At cathode: O_2_ + 2H_2_O + 4e^−^ = 4OH^−^

**Figure 18:**
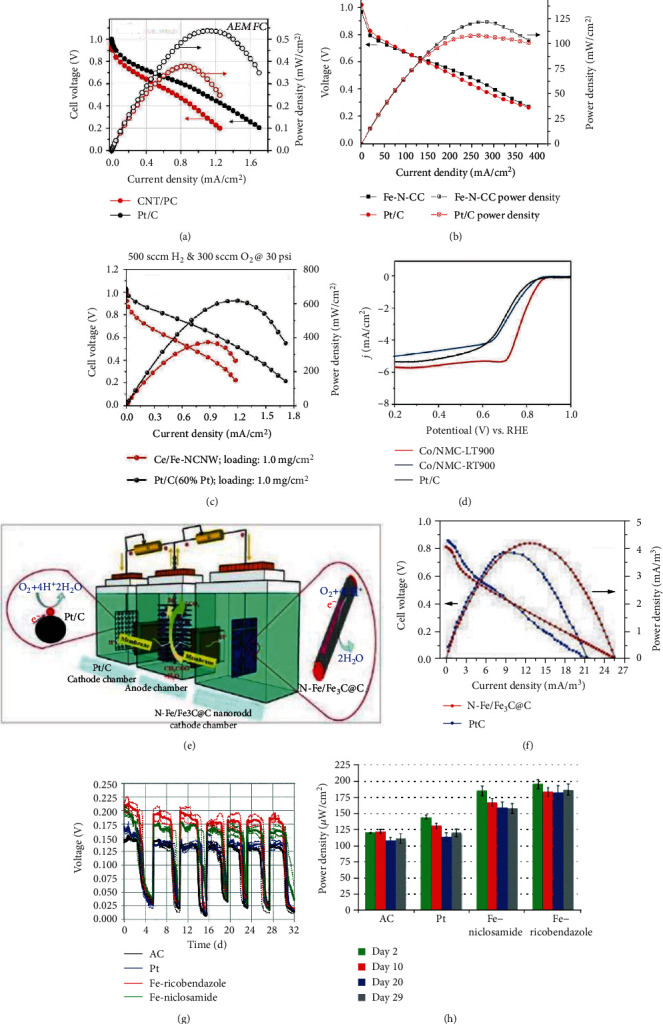
Anion exchange membrane fuel cells and microbial fuel cells with SAECs as the air cathode catalysts in aqueous alkaline and neutral electrolytes, respectively. (a) Comparison of AEMFC performances with different catalysts. Reproduced from [82]. (b) Polarization curve and power density of as-prepared catalysts in AEMFC. Reproduced from [114]. (c) Comparison of AEMFC performance with different cathode catalysts. Reproduced from [193]. (d) LSV curves of as-prepared catalysts in neutral electrolyte. Reproduced from [47]. (e) Schematic illustration of the MFC. Reproduced from [194]. (f) Comparison of MFC performance with different cathode catalysts. Reproduced from [194]. (g) Durability tests of MFCs with different cathode catalysts. Reproduced from [195]. (h) MFC power densities measured at different times. Reproduced from [195].

A Fe-based single-atom catalyst (CNT/PC) by pyrolysis of Fe-porphyrin complexes with a silica protective layer shows high ORR activity in alkaline electrolyte with a half-wave potential of 0.88 V (vs. RHE). As the cathode catalyst of AEMFCs, CNT/PC exhibits outstanding performance, comparable to that of Pt/C catalysts [82]. As shown in Figure 18(a), the current density (at 0.6 V) and peak power density reach 498 mA cm^−2^ and 0.38 W cm^−2^, respectively, which are record high parameters among nonprecious metal electrocatalysts for AEMFCs. Another Fe-based SAEC by embedding Fe-N_*x*_ moieties in the N-doped mesoporous carbon capsules (Fe-N-CC) provided an onset potential of 0.94 V (vs. RHE) and a half-wave potential of 0.83 V (vs. RHE). As the cathode, Fe-N-CC-based AEMFC provides a peak power density of 120 mW cm^−2^, higher than that of commercial Pt/C [114]. The excellent performance of Fe-N-CC was ascribed to (i) abundant nitrogen functional groups within the carbon framework, (ii) the decoration of Fe-N_*x*_ coordination sites, (iii) high specific surface area (1600 m^2^ g^−1^) and porosity composed of uniform mesopores (3.8 nm), (iv) hollow morphology that entails a uniform thin carbon layer (50 nm) greatly reducing diffusion distances, and (v) the existence of graphitic domains enhancing electronic conductivity. By systematically investigating the pore structure of carbon framework, Lee and coworkers fabricated a single-atom Fe-N-C catalyst with optimized hierarchical ordered porous structure from micropores to mesopores and macropores. The Fe-N-C-based AEMFCs provided a current density at 0.6 V and maximum power densities of 518 mA cm^−2^ and 504 mW cm^−2^, respectively, which represents the best performance among AEMFCs reported so far when normalized with catalyst loadings [173]. In a ceria-boosted single-atom Fe-based catalyst, ceria was used to coordinate with Fe atoms, confine Fe atoms in the lattice, and inhibit the migration and agglomeration of isolated Fe atoms during thermal treatments. The resultant Ce/Fe-NCNW contains atomically dispersed Fe up to 4.6 wt%. As the cathode catalyst in an AEMFC, a maximum power density of 496 mW cm^−2^ was achieved [193], as shown in Figure 18(c).

#### 5.4.2. Microbial Fuel Cells (MFCs)

Microbial fuel cells (MFCs) convert chemical energy in organics into electrical energy by the action of microorganisms. MFCs can harvest energy from waste and thus alleviate both energy and environmental problems. In 1911, Potter, for the first time, proposed the idea to generate electricity with microbes [196]. MFCs utilize electroactive bacteria capable of degrading organic compounds and releasing electrons on the conductive anode electrode directly or through mediators or nanowires. Electrons pass through an external circuit from the anode to the cathode where oxygen is reduced, generating electricity. Neutral buffer solutions such as potassium phosphate-buffered solution (PBS) are commonly used electrolytes, which cause inorganic or carbonaceous catalysts suffering from tremendous activation overpotentials resulting in low catalytic activity. The relatively low power density (0.4~1.5 W m^−2^) of air-cathode MFCs caused by high overpotentials and slow reaction kinetics of the oxygen reduction reaction (ORR) hinders wide applications. Recently, some SAECs have been found to show excellent ORR performance under neutral conditions and could be used as the air-breathing cathode catalysts.

As shown in Figure 18(d), a single-atom Co catalyst (Co/NMC-LT900) showed superior ORR activity in O_2_-saturated near neutral electrolyte (0.05 M PBS) to Co cluster or Co nanoparticle catalysts (Co/NMC-RT900) and Pt/C in terms of high kinetic current density and low ORR Tafel slope. The single-atom Co/NMC-LT900 catalyst could be used as the air cathode of MFCs to output a high maximum power density (2550 mW m^−2^) and long durability for more than 820 h [47]. A three-dimensional Fe-N-C catalyst (3D Fe-N-C) featuring with high specific surface area, well-ordered mesopores, highly conductive framework, and atomically dispersed Fe-N sites shows significantly enhanced ORR activity in 0.01 M PBS neutral electrolyte compared to that of commercial Pt/C in terms of positive half-wave potential, stable limiting current, excellent tolerance to methanol, and remarkably long-term durability. As the air cathode catalyst of MFCs, 3D Fe-N-C provided a maximum power density of 3118.9 mW m^−2^ at a high current density of 9980.8 mA m^−2^ [197]. The 3D Fe-N-C-based MFCs could steadily run for more than 250 h in a feed period, which is substantially longer than the Pt-based MFCs. Santoro et al. used a single-atom Fe catalyst (Fe-AAPyr) as the air-breathing cathode of MFCs and systematically investigated the influence of Fe-AAPyr loadings between 0.1 mg cm^−2^ and 10 mg cm^−2^ on the electrochemical performance and the cost MFCs for practical applications [198]. It was found that the MFC performance was gradually enhanced by increasing Fe-AAPyr loadings with power densities increased from 90 *μ*W cm^−2^ to 262 *μ*W cm^−2^. However, the higher the Fe-AAPyr loading is, the higher the MFC cost is. With ricobendazole and niclosamide as the C and N precursors, they further prepared two Fe-based SAECs (Fe-ricobendazole and Fe-niclosamide). As the air cathode of MFCs, Fe-ricobendazole provides an initial peak power density of 204 *μ*W cm^−2^, shows high stability along the 32 days operations, and tolerates to S^2-^ species, which are much superior to those of Pt-based catalysts [195]. Cost analysis shows that the cost of Fe-based catalysts is roughly 31-33 US$ per W produced, approximately 55-fold cost reduction compared to Pt (1770 US$ per W).

By pyrolyzing the mixture of FeCl_3_ and cyanamide at 750°C, a core shell-structured N-Fe/Fe_3_C@C nanorod catalyst containing both single-atom FeN_*x*_ sites and Fe_3_C sites was prepared [194]. N-Fe/Fe3C@C shows remarkably elevated activities and kinetics for ORR in neutral 0.1 M PBS compared with the commercial 10 wt% Pt/C catalyst. As the cathode catalysts of MFCs, N-Fe/Fe3C@C-MFC provides higher current output, higher power density, higher short-circuit current density, and smaller charge transfer resistance and overall internal resistance than those of the Pt/C-MFC. The Coulombic efficiency of N-Fe/Fe3C@C-MFC reaches 57.1% at a *R*_ex_ of 1 *Ω*, nearly twice of the CE (32.38%) of the Pt/C-MFC. The superior catalytic properties of N-Fe/Fe3C@C derived from the synergetic effect from the intrinsic 1D core shell architecture and the strong interaction between conductive carbon shells and core-Fe/Fe3C, which makes the N-doped graphite shells work efficiently along with Fe/Fe3C. To be highlighted, the cost of the raw materials for preparing the N-Fe/Fe3C@C is less than 5% of the Pt/C catalysts.

With 2,6-diaminopyridine as a building-block monomer of a nitrogen-rich network polymer, Zhao et al. prepared a binary CoFe-PDAP SAECs [199], which showed high ORR activity with an onset potential of 0.87 V and a half-wave potential of 0.76 V (vs. RHE) in the PBS neutral electrolyte, much better than single coordination of Co or Fe, possibly because of the increase in coordinated metal species. The limiting current density of CoFe-PDAP was 4.9 mA cm^−2^ at 0.2 V, which is substantially higher than that of Pt/C (3.8 mA cm^−2^ at 0.2 V). As the air-breathing cathode of MFCs, CoFe-PDAP provides short-circuit current density, open-circuit voltage, and maximum power density of 0.53 mA cm^−2^, 0.7 V, and 0.11 0.01 mW cm^−2^, respectively, higher than those for the Pt/C MFCs (0.18 mA cm^−2^, 0.6 V, and 0.05 mW cm^−2^, respectively). Anode and cathode polarization analyses revealed that not only the cathode but also the anode of the CoFe-PDAP MFCs showed higher performance than those in the Pt/C MFCs. The superior activity of the CoFe-PDAP cathode is due to the lessened crossover effects of organics on the ORR activity of CoFe-PDAP compared with Pt/C. The CoFe-PDAP-based MFCs stably operated for more than 1 month.

#### 5.4.3. Proton Exchange Membrane Fuel Cells (PEMFCs)

Proton exchange membrane fuel cells convert the chemical energy released in the electrochemical reaction of hydrogen and oxygen at separated electrodes to electrical energy. The structure of PEMFCs resembles to that of AEMFCs except for the polymer exchange membrane. A stream of hydrogen is supplied and oxidized at the anode. The oxidation half-cell reaction is represented by H_2_ = 2H^+^ + 2e^−^. The generated protons migrate through the polymer electrolyte membrane to the cathode; meanwhile, electrons pass through the external circuit to the cathode. At the cathode, oxygen molecules react with protons and electrons originated from the anode to generate current with water as the only product. The reduction half-cell reaction is represented by O_2_ + 4H^+^ + 4e^−^ = 2H_2_O. The efficiency of PEMFCs is three times higher than conventional internal combustion engines. However, the bottleneck of PEMFCs lies in sluggish ORR kinetics and decay of electrocatalysts under acidic and oxidative conditions. Therefore, electrocatalyst with high activity and long-term durability is urgently needed. At the moment, Pt-based catalysts are regarded as the benchmark ORR electrocatalysts. But high cost and scarce reservation of Pt have limited its large-scale application in PEMFCs. Pt-based catalysts account for 55% of the cost of PEMFC stacks [21]. Hence, to meet the demands for the mass production of PEMFC systems, the guidelines of the United States Department of Energy (DOE) stipulate that the total Pt loading on both electrodes should be no higher than 0.10 g kW^−1^ by 2025. Intensive efforts have been devoted to boosting the mass activity and durability of Pt-based catalysts to meet the DOE target 2025. For example, In O_2_-saturated 0.1 M HClO_4_ electrolyte, a carbon black-supported Pt-based SAEC (Pt_1_-N/BP) with a low Pt loading of 0.4 wt% provided an ORR half-wave potential of 0.76 V, which is close to the commercial Pt/C catalyst with much higher Pt nanoparticle loading of 20%. As the cathode catalyst, Pt_1_-N/BP-based H_2_/O_2_ fuel cell in 100% relative humidity delivers a peak power density of 680 mW cm^−2^ with a low platinum loading of 0.09 mg_Pt_ cm^−2^, corresponding to a platinum utilization of 0.13 g_Pt_ kW^−1^ in the fuel cell [140]. By increasing Pt loading or synergistic interact with atomically dispersed Fe-N-C sites, the platinum utilization could be further reduced to 0.09 g_Pt_ kW^−1^ in H_2_/O_2_ fuel cell [158, 161].

**Figure 19:**
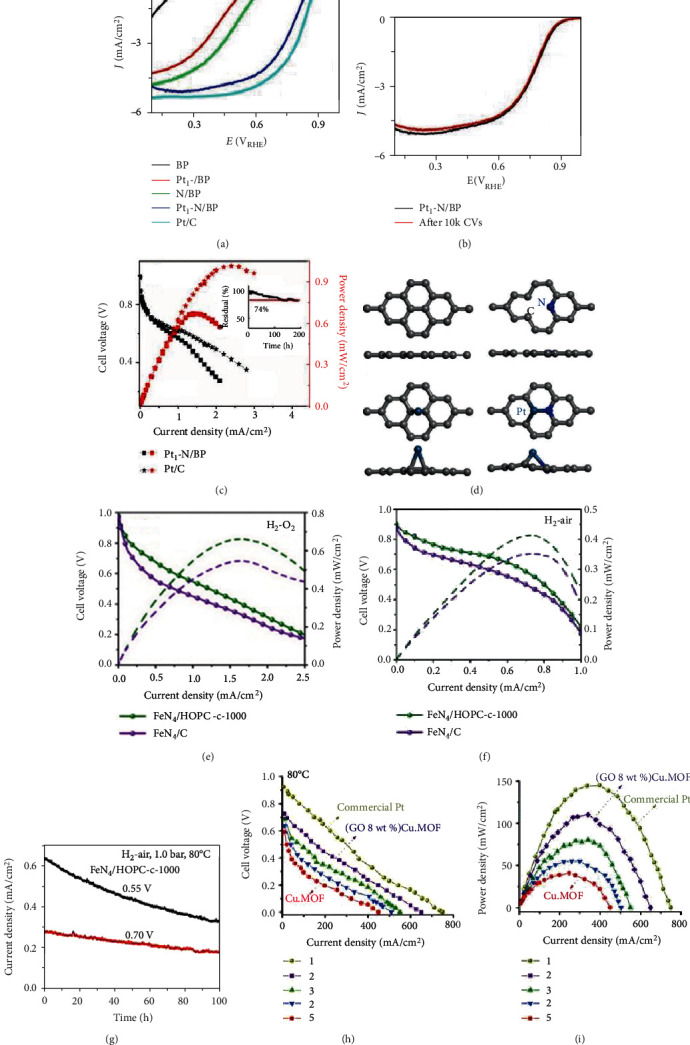
Proton exchange membrane fuel cells with SAECs as the air cathode catalysts in aqueous alkaline and neutral electrolytes, respectively. (a) LSV curves of as-prepared catalysts in acid electrolyte. Reproduced from [140]. (b) Durability test of Pt_1_-N/BP in acid electrolyte. Reproduced from [140]. (c) Performance of H_2_/O_2_ fuel cells with different catalysts. Reproduced from [140]. (d) DFT calculated structures of different samples. Reproduced from [140]. (e) Comparison of PEMFC performance with as-prepared catalysts in 1 bar H_2_/O_2_. Reproduced from [150]. (f) Comparison of PEMFC performance with as-prepared catalysts in 1 bar H_2_-air. Reproduced from [150]. (g) Durability measurements at different potentials. Reproduced from [150]. (h) Comparison of polarization curves for the H_2_/O_2_ fuel cell. Reproduced from [200]. (i) Comparison of power density for the H_2_/O_2_ fuel cell. Reproduced from [200].

On the basis of General Motor's cost accounting report, PEMFC-based engines should decrease the Pt loadings to less than 0.0625 mg cm^−2^ while maintaining power densities higher than 1.0 W cm^−2^ to compete with traditional internal combustion engines in vehicles, which is nearly an impossible challenge to commercialize Pt-based catalysts. In this context, development of Pt-free electrocatalysts meeting the U.S. Department of Energy's cost target ($30 kW^−1^) attracted great attention in very recent years. Among various reported catalysts, noble metal-free SAECs featuring with low cost and encouraging ORR performance are potential candidates to accelerate cathode ORR process in PEMFCs. Atanassov and coworkers fabricated a single-atom Fe catalyst (Fe-CBDZ), which showed high ORR activity and extremely high durability in acidic electrolyte. After 10000 cycles in an oxygen atmosphere, the ORR half-wave potential of Fe-CBDZ maintained more than 94% of its initial activity. In a single membrane electrode assembly (MEA) test, the Fe-CBDZ-based PEMFC showed a remarkably high open-circuit voltage of 1 V, which is the second best performance ever reported with no IR correction. MEA provided high current density up to 700 mA cm^−2^ at 0.6 V and 120 mA cm^−2^ at 0.8 V. SAECs prepared by pyrolyzing Fe-doped ZIF-8 show comparable ORR activity to commercial Pt/C, but the micropores limit rapid mass transfer. Qiao et al. prepared single-atom Fe catalysts (FeN_4_/HOPC) by embedding atomically dispersed FeN_4_ sites in an ordered hierarchical porous structure [150]. The optimal FeN_4_/HOPC-c-1000 catalyst exhibits outstanding performance with a half-wave potential of 0.80 V in 0.5 M H_2_SO_4_ solution, only 20 mV lower to that of commercial Pt/C (0.82 V). As shown in Figure 19(e), in a real H_2/_O_2_ PEMFC, FeN_4_/HOPC-c-1000-based cathode provides an open-circuit voltage of 0.98 V, a current density of 0.75 A cm^−2^ at 0.6 V, and a peak power density of 0.42 W cm^−2^, superior to those of FeN_4_/C without an optimized pore structure.

The activity goal set by US DOE for Pt-free catalysts in PEMFCs is to achieve a current density of 0.044 A cm^−2^ under 1.0 bar H_2_/O_2_ at 0.88 V_iR-free_ in 2018 and at 0.9 V_iR-free_ in 2020. The DOE 2018 target has been realized due to the great progress made by Wan's group. They prepared a Fe-based SAEC (TPI@Z8(SiO_2_)-650-C) with a concave-shaped external surface structure and high Fe-N_*x*_ density. As the cathode catalyst, TPI@Z8(SiO_2_)-650-C-based PEMFCs provide a current density of 0.047 A cm^−2^ at 0.88 V_iR-free_ under 1.0 bar H_2_/O_2_ [182], achieving the DOE 2018 target. Furthermore, the peak power density of 1.18 W cm^−2^ and current density of 129 mA cm^−2^ at 0.8 V_iR-free_ are achieved under 2.5 bar H_2_/O_2_ and 1.0 bar H_2_-air, respectively. The unprecedented PEMFC performance could be attributed to the rationally designed TPI@Z8(SiO2)-650-C that enables highly efficient utilization of the densely Fe-N_4_ sites and remarkably enhances mass transport. Moreover, quantitative analysis showed that the active-site density is the predominant parameter in governing the fuel cell activity of single-atom Fe-N-C catalysts, while the external surface area and mesoporous structure play significant part in elevating active-site density and enhancing the mass transport.

Fe-based SAECs show high intrinsic ORR activity and huge potential to substitute Pt-based catalysts. But deactivation and decay of Fe-based SAECs in acid electrolytes greatly limited their commercial applications in PEMFCs, owing to the Fenton reaction. The generated free radicals would oxidize the carbon support, destroy the active sites, and even degrade the proton exchange membrane. In contrast, Co- and Mn-based ions are inactive for the Fenton reaction, which makes their corresponding SAECs potential candidates as durable cathode catalysts for PEMFCs. Wang et al. prepared a Co-based SAECs (20Co-NC-1100) by pyrolysis of Zn/Co bimetallic ZIF [63]. Atomically dispersed 20Co-NC-1100 showed high ORR activity in 0.5 M H_2_SO_4_ with a half-wave potential of 0.8 V (vs. RHE), comparable to PANI-derived Fe-N-C catalyst and 60 mV lower than Pt/C (60 *μ*g Pt cm^−2^). 20Co-NC-1100 also exhibited respectable stability in acidic media with only a loss of 30 mV in *E*_1/2_ after 10000 cycles, compared to a loss of 80 mV for Fe-N-C after 5000 cycles. As the membrane assembly electrodes (MEAs) with 20Co-NC-1100 loading of 4.0 mg cm^−2^ (approximately 0.08 mg_Co_ cm^−2^) of a H_2_/O_2_ fuel cell, an open-circuit voltage of 0.95 V is achieved, which is comparable to single-atom Fe-N-C catalyst. The H_2_/O_2_ fuel cell using 20Co-NC-1100 as cathode catalysts showed the peak power density of 0.56 W cm^−2^. The peak power density achieved 0.28 W cm^−2^ in H_2_/air fuel cell for 20Co-NC-1100, much higher than single-atom Fe-N-C catalyst. The durability test for the 20Co-NC-1100 at 0.7 V in H_2_/air fuel cell shows that at initial stage up to 30 h, there are insignificant losses (less than 15 mV) at all current density ranges, and a 100 h continuous operation eventually results in a loss around 60 mV, indicating significant enhancement of performance durability. By increasing the atomically dispersed CoN_4_ density with surfactant-assisted confinement, they further improved the half-wave potential of Co-based SAECs (Co-N-C@F127) to 0.84 V in 0.5 M H_2_SO_4_ [62], which approaches state-of-the-art Pt/C catalysts. The Co-N-C@F127 also demonstrates excellent stability with a loss of only 40 mV in *E*_1/2_ after 30000 potential cycles from 0.6 to 1.0 V. At moderate voltages (0.5-0.7 V) typical of PEMFC operation, the Co-N-C@F127 could generate a peak power density of 0.87 W cm^−2^. It has been found that Mn atoms could catalyze the graphitization of the organic precursor in the carbonization process and thus Mn dopants in the carbon matrix enhance the stability of resultant nanocarbon skeleton, which promotes the stability of Mn-based SAECs. Li and coworkers prepared Mn-based SAECs (20Mn-NC-second) with high density of MnN_4_ sites [154]. In comparison with the Fe-N-C catalyst prepared in the same procedure, 20Mn-NC-second exhibited comparable ORR activity with a half-wave potential of 0.80 V in the acidic electrolyte and much better durability with only 17 mV shift in *E*_1/2_ after 30000 potential cycles in O_2_-saturated 0.5 M H_2_SO_4_ solution. The microstructure and morphology of the carbon framework in the 20Mn-NC-second catalyst maintained well after the potential cycling tests. As a cathode catalyst in MEAs, an open-circuit voltage of 0.95 V is achieved using H_2_ and O_2_. The 20Mn-NC-second cathode could provide a peak power density of 0.46 W cm^−2^, which is inferior to the single-atom Fe-N-C catalyst especially in the kinetic range. But 20Mn-NC-second catalyst showed much better stability than Co- and Fe-based SAECs under real PEMFC operating condition tests at 0.7 V for 100 h. The 20Mn-NC-second catalyst acts as one of the most durable SAECs in acidic electrolytes.

Cu-based complexes exhibit biomimetic chemistry with oxygen molecules, such as the reductive activation of O_2_ in enzymes and the protein laccase. To date, most Cu-based complexes have been studied in alkaline electrolyte due to their corrosion and instability in acid media. To address the stability issue in acid, Jahan et al. designed a GO-incorporated Cu-MOF hybrid single-atom catalyst ((GO 8 wt%)Cu-MOF) [200], which can coordinate with two strong electronegative ligands based on oxygen and nitrogen functional groups, thus leading to an improvement in the framework stability, especially when it is encapsulated by GO in acid media. As the air cathode of H_2_ and O_2_ fuel cell, (GO 8 wt%)Cu-MOF provides an open-circuit voltage of 0.73 V and a maximum power density of 110.5 mW cm^−2^ (approximately 76% that of Pt/C catalyst).

Although great progress has been made on Fe-, Co-, Mn-, and Cu-based SAECs to increase intrinsic ORR activity and improve durability in acidic electrolytes, their commercial applications in PEMFCs have not been realized. In particular, their output performance and durability in PEMFCs are still much lower than Pt-based electrocatalysts. Further optimization of SAECs and clarification of deactivation mechanisms are urgently needed to impel commercialization of SAEC-based PEMFCs.

## 6. Conclusions and Prospects

Single-atom electrocatalysts are of great significance for speeding up sluggish kinetics of oxygen reduction reactions with high efficiency and low cost. Quantum size effect, low-coordination environments, and strong bonding between atomically dispersed metal atoms and supports enable SAECs showing fascinating features including maximum atomic utilization, high activity, favorable charge transfer kinetics, and excellent durability. Single-atom electrocatalysts show superior or comparable ORR activity to commercial Pt/C catalysts in alkaline or acidic electrolytes and have been proven to be powerful in typical 2e^−^ and 4e^−^ ORR processes as well as applications in H_2_O_2_ production, metal-air batteries, and low-temperature fuel cells. Encouragingly, in very recent years, understanding of SAECs including synthetic methods, characterization technologies, theoretical calculations, and the structure-performance relationship has made great progress. However, following challenges still need to be addressed:

- SAECs offer ideal models with uniform active-site structure to quantitatively investigate the intrinsic activity, in situ detect the activation of intermediates, and establish the structure-performance relationship. Finely modulating the coordination environment of isolated metal atoms is highly desirable and challenging to obtain SAECs with uniform active-site structures
- Advanced in situ or operando characterization technologies are necessary to unravel the real ORR catalytic process over SAECs. Currently, the atomic structure of the active metal center in SAECs is characterized mainly based on the linear square fitting of ex situ XAS spectra. Detailed in situ or operando measurements should be performed to illustrate the real interaction between the central metal and oxygen-containing species under working potential conditions. In addition, some uncertainty exists on the quantitative and accurate identification of the coordinated atoms. For example, it is difficult to distinguish the XAS signals from N(C) or N(O) in the first coordination shell.
- The synergistic effect between two adjacent metal atoms could improve the ORR performance in terms of intrinsic activity and durability. The synthesis of SAECs with multiple, correlative, and uniform metal centers is of great challenge and significance.
- Besides activity and selectivity, durability is another important parameter for SAECs in practical applications, especially in acidic electrolytes. Acidic electrolytes might cause metal centers being exchanged by protons, degrade the carbon support, and result in significant activity loss, especially for the single-atom Fe-N-C catalysts due to the undesired Fenton reaction. Therefore, developing advanced support materials with high conductivity and corrosion resistance are highly desired.
- The mass and electron transports during ORR process are closely related with the limit current density and the peak power density, which are mainly controlled by the pore structure of the support. Designing support materials with unique structure, hierarchical porosity, and high specific surface to enhance the transport process and increase the accessible active-site numbers is of great effectiveness.
- Currently reported synthetic methods for SAECs require expensive precursors and equipment. Achieving simple, low-cost, and large-scale synthesis of SAECs with high metal loadings is still a major challenge that limits their practical applications.

It is expected that when the abovementioned challenges have been realized, the highly efficient, green, and economic electrochemical energy conversion and storage systems will power our daily life and improve our energy structures
